# Interactions of Viral Proteins from Pathogenic and Low or Non-Pathogenic Orthohantaviruses with Human Type I Interferon Signaling

**DOI:** 10.3390/v13010140

**Published:** 2021-01-19

**Authors:** Giulia Gallo, Grégory Caignard, Karine Badonnel, Guillaume Chevreux, Samuel Terrier, Agnieszka Szemiel, Gleyder Roman-Sosa, Florian Binder, Quan Gu, Ana Da Silva Filipe, Rainer G. Ulrich, Alain Kohl, Damien Vitour, Noël Tordo, Myriam Ermonval

**Affiliations:** 1 Unité des Stratégies Antivirales, Institut Pasteur, 75015 Paris, France; giulia.gallo@pasteur.fr (G.G.); noel.tordo@pasteur.fr (N.T.); 2 Ecole Doctorale Complexité du Vivant, Sorbonne Université, 75006 Paris, France; 3 UMR 1161 Virologie, Anses-INRAE-EnvA, 94700 Maisons-Alfort, France; gregory.caignard@vet-alfort.fr (G.C.); damien.vitour@vet-alfort.fr (D.V.); 4 BREED, INRAE, Université Paris-Saclay, 78350 Jouy-en-Josas, France; karine.badonnel@inrae.fr; 5 Institut Jacques Monod, CNRS UMR 7592, ProteoSeine Mass Spectrometry Plateform, Université de Paris, 75013 Paris, France; guillaume.chevreux@ijm.fr (G.C.); samuel.terrier@ijm.fr (S.T.); 6 MRC-University of Glasgow Centre for Virus Research, Glasgow G61 1QH, UK; agnieszka.szemiel@glasgow.ac.uk (A.S.); Quan.Gu@glasgow.ac.uk (Q.G.); ana.dasilvafilipe@glasgow.ac.uk (A.D.S.F.); alain.kohl@glasgow.ac.uk (A.K.); 7 Unité de Biologie Structurale, Institut Pasteur, 75015 Paris, France; gleyder.roman-sosa@vetmed.uni-giessen.de; 8 Friedrich-Loeffler-Institut, Institute of Novel and Emerging Infectious Diseases, 17493 Greifswald-Insel Riems, Germany; binderflorian@aol.com (F.B.); rainer.ulrich@fli.de (R.G.U.); 9 Institut Pasteur de Guinée, BP 4416 Conakry, Guinea

**Keywords:** orthohantavirus, interferon response, nonstructural protein, glycoprotein, Puumala virus, Tula virus, Prospect Hill virus

## Abstract

Rodent-borne orthohantaviruses are asymptomatic in their natural reservoir, but they can cause severe diseases in humans. Although an exacerbated immune response relates to hantaviral pathologies, orthohantaviruses have to antagonize the antiviral interferon (IFN) response to successfully propagate in infected cells. We studied interactions of structural and nonstructural (NSs) proteins of pathogenic Puumala (PUUV), low-pathogenic Tula (TULV), and non-pathogenic Prospect Hill (PHV) viruses, with human type I and III IFN (IFN-I and IFN-III) pathways. The NSs proteins of all three viruses inhibited the RIG-I-activated IFNβ promoter, while only the glycoprotein precursor (GPC) of PUUV, or its cleavage product Gn/Gc, and the nucleocapsid (N) of TULV inhibited it. Moreover, the GPC of both PUUV and TULV antagonized the promoter of IFN-stimulated responsive elements (ISRE). Different viral proteins could thus contribute to inhibition of IFNβ response in a viral context. While PUUV and TULV strains replicated similarly, whether expressing entire or truncated NSs proteins, only PUUV encoding a wild type NSs protein led to late IFN expression and activation of IFN-stimulated genes (ISG). This, together with the identification of particular domains of NSs proteins and different biological processes that are associated with cellular proteins in complex with NSs proteins, suggested that the activation of IFN-I is probably not the only antiviral pathway to be counteracted by orthohantaviruses and that NSs proteins could have multiple inhibitory functions.

## 1. Introduction

Orthohantaviruses (family *Hantaviridae*, subfamily *Mammantavirinae*, genus *Orthohantavirus*) belong to the *Bunyavirales* order, according to International Committee on taxonomy of viruses (ICTV) taxon classification update (https://talk.ictvonline.org/taxonomy/). In contrast to most of the viruses in the order, orthohantaviruses are not arboviruses and they are hosted by rodents and small insectivorous mammals (moles, shrews). They have co-evolved for thousands of years with their animal reservoir and exhibit exquisite natural host specificity with these natural hosts [1,2]. Along with their reservoir, rodent-borne orthohantaviruses are distributed all over the world and some of them are responsible for disease clusters or outbreaks, which makes these emerging and re-emerging viruses of great public health concern [3].

Orthohantaviruses circulate among their natural hosts without provoking any detectable symptoms. Although predominantly infecting lungs and kidneys, these viruses are present in many tissues and they can persist in their host reservoirs during their entire life. Occasional transmission to humans occurs by inhalation of viruses present in aerosolized excreta of infected rodents and can lead to different pathologies. Hemorrhagic fever with renal syndrome (HFRS) is mainly reported in Europe and Asia, while hantavirus cardiopulmonary syndrome (HCPS) occurs exclusively in the Americas. However, these diseases share common features, such as an alteration of vascular permeability and thrombocytopenia. Because orthohantaviruses are not cytopathic, these pathologies are thought to be caused by a burst of cytokines that are secreted by infected endothelial cells or by cells of the innate immune system recruited at the site of infection [4]. Virus infected cells usually react by producing interferon (IFN), which, in turn, upon binding to IFN receptors, activates the JAK/STAT pathway and a cascade of events, leading to the transcription of genes encoding antiviral proteins. Therefore, to replicate, disseminate in an organism, and propagate, viruses have evolved different strategies to antagonize these cellular responses [5]. On the one hand, orthohantaviruses interact with the innate immune system by exacerbating the innate response at the level of targeted organs, while, on the other hand, they must counteract the IFN antiviral pathway [6,7]. 

The induction of IFN has been described in patients with hantaviral diseases as well as in infected primary cells or cell lines of human origin. The expression levels of both proteins and mRNA specific of different types of IFN and of IFN-stimulated genes (ISG) with antiviral activities, such as MxA or ISG 56 [8,9], have been measured. For instance, the induction of type I IFN (IFN-I, α and β) has been described in human endothelial cells and macrophages [9,10] and type III IFN (IFN-III, λ) in VeroE6 monkey cells [11]. It has been shown that higher levels of IFN-I were induced in A549 and HuH7 cells that were infected with non-pathogenic Prospect Hill orthohantavirus (PHV) as compared to pathogenic Hantaan orthohantavirus, HTNV [8]. Differences also appear in the kinetics of infection when comparing pathogenic Andes orthohantavirus (ANDV) and non-pathogenic PHV. While both of the viruses can inhibit IFN signaling, PHV provokes an earlier IFN induction than ANDV [12]. Although no clear-cut results have been obtained to discriminate pathogenic and non-pathogenic viruses, it is thought that a delayed IFN-I antiviral state could be associated with the severity of HFRS [13]. However, orthohantaviruses seem to be weak inducers of IFN-I and IFN-III, both in vivo and in vitro [4].

In the case of other bunyaviruses, nonstructural proteins that are encoded by the S (NSs) or the M (NSm) segments have been described to act as inhibitors of the antiviral response, thereby promoting viral replication. In particular, NSs protein antagonizes IFN production or its downstream signaling effectors in different ways depending on the virus, even when they belong to the same family, as documented for phleboviruses [14]. For example, the NSs protein of the Rift Valley fever virus (RVFV) interferes in the nucleus with the IFN promoter, while the NSs protein of severe fever with thrombocytopenia syndrome virus (SFTSV) may act at different levels of the IFN pathway by sequestering transcription factors, such as the IFN regulatory factor 3, IRF3 [15], or the signal transducer and activator of transcription 1, STAT1, in inclusion bodies [16,17]. Of note, despite their common name, NSs proteins are highly divergent and they use various coding strategies across families. Therefore, NSs proteins might cover a large panel of functions.

The genome of orthohantaviruses is composed of three segments of single stranded RNA of negative polarity encoding four structural proteins. The S segment encodes the nucleocapsid (N), the M segment encodes the glycoprotein precursor (GPC) of the envelope Gn and Gc proteins, and the L segment encodes the RNA-dependent RNA polymerase. Despite this small number of proteins, orthohantaviruses can still manipulate cellular pathways in order to efficiently replicate and disseminate. The inhibition of antiviral defense is mainly considered to be associated to the structural proteins N and Gn [18]. 

In contrast to other bunyaviruses, the majority of orthohantaviruses do not encode NSs proteins. Nevertheless, a few orthohantaviruses hosted by rodents of the Arvicolinae subfamily, such as the pathogenic Puumala virus (PUUV), the low-pathogenic Tula (TULV) from Europe, and the non-pathogenic virus PHV from North America, could express NSs protein. Indeed, a short open reading frame (ORF) encoding a protein of 90 amino acid (aa) residues that were obtained from a +1 nucleotide (nt) frame shift is found approximately 40 nt downstream of the N-ORF start. An even shorter 63 aa long putative NSs is produced by leaky scanning in the same region of S segments of orthohantaviruses infecting rodents of the Sigmodontinae and Neotominae subfamilies [19,20] found in the American continent, such as ANDV and Sin Nombre virus (SNV). The existence of these putative NSs proteins has been highlighted by indirect studies due to the lack of reverse genetics system for hantaviruses, but their exact function has yet to be clarified [21]. 

Here, we aimed to compare the impact on human antiviral response of the structural proteins, N, and the cytosolic domain of Gn (GnCT) expressed under different forms or in a glycoprotein full-length context, as well as the nonstructural NSs proteins. Viral proteins were produced from both pathogenic and low or non-pathogenic orthohantaviruses. We expressed these proteins individually or in combination by the transfection of corresponding plasmid constructs to test their effect on different elements of the IFN cascade in reporter luciferase (Luc) assays. Both retinoic acid-inducible gene I (RIG-I) induced IFNβ-promoter activation and ISRE-promoter activation by recombinant IFNβ were evaluated. This study was extended to the expression of genes that are specific of antiviral response detected during viral infection by performing transcriptomic and quantitative reverse transcription-polymerase chain reaction (RT-qPCR) analyses in infected A549 cells. Moreover, in order to obtain better insight into NSs activities, we performed mass spectrometry (MS) analysis of cellular proteins in complex with NSs protein of PUUV and TULV that is expressed in transfected human HEK293T cells.

## 2. Materials and Methods 

### 2.1. Cells 

VeroE6, an African green monkey kidney cell line, kindly provided by A. Rang (Charité, Berlin, Germany), and HEK293T, human embryonic kidney cells transformed by SV40 large T antigen, were grown in DMEM/glutamax culture medium that was supplemented with 10% heat-inactivated fetal bovine serum (DMEM/10% FBS, Biosera, Nuaille, France). Medium and additives were purchased from Gibco, except when indicated. HuH7, a human hepatocarcinoma cell line was cultured in DMEM/10% FBS that was supplemented with non-essential amino acids and 1 mM sodium pyruvate. The human alveolar adenocarcinoma A549 cell line (ATCC^®^ CCL185^™^) was cultured in 10% FBS enriched F12-K + L-glutamine medium (Corning, MA, USA). All of the cell lines were maintained at 37 °C in 5% CO_2_ atmosphere and regularly controlled as being mycoplasma free using the Mycoalert kit (Lonza, Basel, Switzerland) according to the manufacturer.

### 2.2. Viruses

PUUV Sotkamo (wild type strain and its related variant clone PUUV #9 [22]), as well as PHV, were a kind gift from A. Rang (Charité, Berlin, Germany). PUUV#9 is impaired in full-length NSs expression, due to a mutation in its coding sequence introducing a stop codon at position 21 of NSs amino acid sequence. Here, it is referred to as PUUVΔNSs. The TULV strain Lodz was provided by A. Vaheri and TULV strain Moravia by A. Plyusnin (Helsinki University, Helsinki, Finland) [23]. The NSs of TULV Moravia possesses shortened NSs, due to a mutation in its coding sequence introducing a stop codon at position 15 of its amino acid sequence, similarly to PUUVΔNSs. 

Viral stocks were produced on VeroE6 cells. Briefly, 3 × 10^6^ cells were seeded in T75 flasks in DMEM/10% FBS. The following day, medium was removed, and the cells were incubated at 37 °C for 1 h with 2 mL of diluted virus in DMEM/5% FBS, at a final multiplicity of infection (MOI) of 0.1. Subsequently, 20 mL of this same medium were added. Supernatants were then collected six days (TULV) or seven days (PUUV and PHV) post infection, aliquoted, and then frozen at −80 °C until use. Viral titers of PUUV, PUUVΔNSs, and TULV Lodz stocks were quantified as being around 2–5 × 10^5^ infectious particles/mL (i.p./mL), and around 3–5 × 10^7^ i.p./mL and 2 × 10^6^ i.p./mL for TULV Moravia and PHV, respectively.

### 2.3. Immunofluorescence Labelling

VeroE6 cells were seeded on glass coverslips in 24-wells plates at 2 × 10^4^ cells/well before transfection or infection. Prior to the transfection, the cells were washed once with DMEM and 0.5 mL of DMEM/well was added. Polyethylenimine (PEI, Sigma–Aldrich, Lyon, France), the agent used for the transfection, was mixed with plasmids at a ratio 4:1 in 200 μL of DMEM. The transfection mix was incubated 20 min at room temperature (RT), before being added onto cells and then incubated for 3 h at 37 °C. The mix was then removed and 1 mL/well of complete medium was added. In some cases, the transfections were performed using the jetPRIME^®^ (Polyplus transfection, Illkirch, Fance) reagent, which was routinely used for the luciferase assay, as described below. For infection, the cells were incubated for 1 h with 150 μL of virus diluted in DMEM/5% FBS before adding 1 mL of the same medium. After transfection or infection, the cells were washed with PBS and fixed at different time points with 3.7% formaldehyde for 15 min at RT. The fixed cells were incubated with PBS/20 mM glycine for 15 min at RT and then permeabilized with TritonX100 at 0.5% in PBS for 5 min. For immunodetection, the cells were incubated with primary antibodies diluted in PBS with 0.5% Tween 20 (PBS-T) containing 1% BSA, for 1 h, at RT. We used mouse monoclonal antibodies (mAb): A1C5 (www.antibodies-online.com) targeting the N of different hantaviruses, anti-flag antibody M2 (Sigma–Aldrich), and anti-streptag clone 661 (Novus). The rabbit anti-Gn antiserum was prepared in the laboratory by the immunization of a rabbit (animal facility, ANSES, Maisons-Alfort, France) with purified Gn from PUUV, adjuvanted with Montanide^TM^ ISA70 VG (kind gift of Seppic, Maisons-Alfort, France). After washing with PBS-T, the cells were incubated for 1 h at RT with goat anti-mouse or anti-rabbit immunoglobulin G (IgG), coupled to Alexa Fluor^TM^ 488 or 555 (Invitrogen). The cells were washed and mounted in Fluoromount DAPI-G (Southern Biotechnology, Birmingham, AL, USA). When not indicated, reagents were obtained from Sigma–Aldrich. The samples were then analyzed in epifluorescence with a microscope equipped with excitation/emission filters (DMLB microscope, Leica, Nanterre, France), or using a Zeiss ApoTome inverted widefield microscope that was equipped with ApoTome grids allowing for global fluorescence and optical sections imaging (UTech bioimaging, Institut Pasteur, Paris, France). 

### 2.4. Plasmid Constructions for Luciferase Assay

The Gateway™ technology (Invitrogen, Waltham, MA, USA) was used in order to clone viral-ORFs in different expression plasmids. Briefly, genes of interest were inserted in the donor plasmid pDONR207 (Invitrogen), sequenced (Eurofins, Paris France), and then transferred in different destination vectors, such as pCiNeo−3xFlag, pStrepTag, peGFP-N1, peGFP-C1, and pmCherry, which allowed for expressing fusion proteins by fusing the coding sequences of the genes in frame with a tag in their 5′ end or 3′ end. The plasmid construct backbones were obtained from Y. Jacob (Institut Pasteur, Paris, France) [24,25]. N ORFs of PUUV Sotkamo and PHV, as well as the different variants of GnCT, i.e., TM1-GnCT, GnCT-TM2, and GnCT from PUUV Sotkamo and TULV Moravia were purchased as synthetic genes from GeneCust. In the case of TULV Moravia, S and M segments in pCMV (a kind gift from A. Plyusnin) were used to amplify N, NSs, and GPC coding sequences for cloning. PHV NSs and the different forms of PHV GnCT were cloned from viral RNA by reverse transcription while using Titan One-Tube RT-PCR kit (Roche), according to the manufacturer’s instructions. The different N, NSs, and GnCT coding sequences of the three viruses were then cloned in the Gateway system by PCR while using specific primers that were flanked by attB sequences. Reactions were performed in a thermal cycler (G-Storm G1, Gene Technologies), as follows: one cycle of 30 min at 50 °C, then 2 min at 94 °C, 35 cycles of 30 s at 94 °C, 30 s at 60 °C, 45 s at 68 °C, and a final elongation step of 7 min at 68 °C. Table S1a presents the list of the primers. 

Plasmids encoding the full-length GPC of PUUV Sotkamo and TULV Moravia with a StrepTag in amino-terminus (N-ter) were obtained, as described [26]. PUUV GPC-eGFP, with an enhanced green fluorescent protein (eGFP) inserted between the transmembrane (TM) and the cytosolic tail (CT) domains of Gn, was generated while using the Gibson assembly master mix (New England Biolabs), according to the instructions of the manufacturer. The plasmids pHan-2 and pME-8 were used as templates to amplify the tagged PUUV GPC coding sequence [27] and the insert, respectively. 

Of note, the genes that were used to activate the IFN pathway were also available in plasmids expressing different tags and they were also a kind gift of Yves Jacob (Institut Pasteur, Paris). RIG-I, MDA5 (melanoma differentiation-associated protein 5), TBK1 (TANK binding kinase 1), and IRF3 possess a Flag tag, and IKKε (inhibitor of nuclear factor kappa-B kinase subunit epsilon) a V5 tag.

### 2.5. Luciferase Assay 

HEK293T cells that were seeded at a density of 2.5 × 10^6^ cells/well in 24-well plates were transfected 24 h later with different plasmids using the jetPRIME^®^ reagent (Polyplus). Briefly, cells were transfected with a mix containing 100 ng of firefly luciferase reporter plasmid under the control of either IFNβ or ISRE promoters (IFNβ-Luc or ISRE-Luc), 10 ng of *Renilla* luciferase reporter plasmid (pCMV-RL), used for the normalization of the assay and 100 ng of plasmid encoding intermediate proteins of the IFN signaling cascade i.e., either the constitutively active N-ter part of RIG-I, the full-length sequences of MDA5 helicase, IKKε and the serine/threonine kinase TBK1, or the mutated and constitutively activated form of IRF3, IRF3-5D. Subsequently, 3 to 300 ng of plasmid encoding viral proteins were also added to the transfection mix, according to the experimental procedure. For the mock treated controls, the amount of plasmid carrying the viral protein was replaced by an equal amount of the corresponding empty backbone plasmid. 

After 24 h, the cells were lysed in Passive Lysis Buffer (Promega, Madison, WI, USA) and 50 μL of lysate were mixed with an equal volume of luciferases’ substrates mix (Bright-Glo and *Renilla*-Glo Luciferase Assay System, Promega) in separated wells. Light emission due to luciferase activity was measured for 5 s/well in a luminometer reader plate (Centro LB 960, Berthold, Baden Württemberg, Germany).

The inhibitory activity of viral proteins on activated IFNβ-Luc was then measured as a reduction in firefly light emission. In the case of ISRE, activation was induced by the incubation of the cells with recombinant IFNβ for 18 h before luminescence measurement. The percentage of activity of each viral protein on the IFN pathway was calculated as the ratio between the absolute chemiluminescent values of firefly luciferase signal (FF_1_) normalized to *Renilla* luciferase signal (FF_1_/Ren_1_) and the normalized luciferase activity of the backbone plasmid exogenously activated (FF_+_/Ren_+_), as follows: % of luciferase activity = ((FF_1_/Ren_1_)/(FF_+_/Ren_+_)) × 100.

The experiments were performed in triplicates for each condition and then repeated at least three times. Statistical significance of differences was calculated while using *nparcomp* package in R applying Tukey comparison. The data are shown as mean ± standard deviation (SD), and * represents significant differences compared to controls of *p*-value < 0.005.

### 2.6. Immunoblotting Analysis 

Cells infected, or transfected, using the PEI agent, as described above, were lysed with NET buffer (150 mM NaCl, 50 mM Tris-HCl pH 7.5, 5 mM EDTA, 0.5 mM AEBSF protease inhibitor) supplemented with 1% Triton, cocktail of protease inhibitor (cOmplete™, Roche), and phosphatase inhibitors (PhosSTOP™, Sigma) for 5 min at RT, and the post nuclear supernatants from cell lysates (cytosolic fractions) were obtained by centrifugation at 13,000 rpm for 15 min at 4 °C and then kept frozen at −80 °C. In order to separate cytosolic and nuclear phases, transfected VeroE6 cells were recovered through scratching and treated using the cytoplasmic buffer (10 mM Tris-HCl pH 8, 5 mM EDTA, 0.5 mM EGTA, 0.5 mM AEBSF, 0.25% TritonX100). Pellets of nuclei were suspended in nuclear lysis buffer (10 mM Tris-HCl pH 7.4, 1 mM EDTA, 0.5 mM AEBSF, 1 mM DTT, 8 M urea) and boiled at 95 °C for 10 min They were then centrifuged at 13,000 rpm for 15 min at 4 °C and the nuclear extracts were recovered and kept frozen at −80 °C. 

Quantification of the amount of proteins in the different lysates was performed with Micro BCA Protein Assay kit (Thermo Scientific, Waltham, MA, USA). Proteins that were diluted in Laemmli buffer were run on sodium dodecylsulphate (SDS) 4–15% gradient polyacrylamide gels (Bio-Rad, Hercules, CA, USA) under denaturing conditions. They were transferred onto nitrocellulose membrane (GE Healthcare). The transfer was controlled by Ponceau S staining (Sigma–Aldrich) of the membrane, for 15 min at RT under shaking. After saturation for 1 h, at RT in PBS-T that was supplemented with 5% skimmed milk, the nitrocellulose membrane was incubated under shaking overnight at 4 °C with primary antibodies diluted in the same buffer. The membranes were then washed with PBS-T at RT before adding anti-mouse or anti-rabbit IgG secondary antibodies (Southern Biotechnology) coupled to horseradish peroxidase (HRP) for 1 h, at RT or directly reacted for 1 h with anti-Flag-HRP (Sigma Aldrich) or anti-StrepTag-HRP (IBA Lifesciences). The membranes were then incubated with a peroxidase substrate (ECL Prime GE Healthcare) and light emission was revealed by exposure to X-ray films (Hyperfilm ECL, GE Healthcare). 

### 2.7. In Silico Analysis and Site Directed Mutagenesis 

In silico analysis of NSs nucleolar localization (NoLS) motif was performed while using the NoD software, an online-available algorithm (http://www.compbio.dundee.ac.uk/www-nod/) developed by G.J. Barton [28]. The algorithm of trained artificial neural network allowed for the prediction of NoLS regions, characterized by a predetermined threshold of 0.8. In parallel, the prediction of intrinsically disordered region of NSs proteins was obtained by amino acid sequence analysis with the PONDR^®^ online software (http://www.pondr.com/).

Site directed-mutagenesis was performed on 50 ng of tagged-NSs plasmids, while using PFU Ultra HF polymerase (Agilent, Santa Clara, CA, USA), in order to validate predicted nucleolar addressing sequence identified in NSs protein. Table S1b lists the primers designed on Agilent website (https://www.agilent.cm/store/primerDesignProgram.jsp). The reactions were carried out in a thermal cycler, as follows: denaturation at 95 °C for 30 s followed by 18 cycles each for 30 s at 95 °C, 1 min at 55 °C, and 5 min at 68 °C. To get rid of the plasmid used as DNA matrix, the amplified products were treated with *DpnI* restriction enzyme for 2 h at 37 °C. Plasmids were then amplified in DH5α strain of *Escherichia coli* (Invitrogen).

### 2.8. RNA-Seq Data Analysis of A549 Cells Infected by PUUV 

The A549 cells were plated at 1.5 × 10^4^ cells per well of 12-well microplates and infected 24 h later with PUUV at MOI 1 in DMEM/5% FBS, or left uninfected. Two wells were prepared per condition and three independent experiments were performed. RNA was extracted at day 5 post-infection with 200 μL per well of TRI Reagent^®^ (Sigma), each sample was spun at 5000 rpm for 10 min at 4 °C. The aqueous phase was precipitated with an equal volume of isopropanol. RNA was then precipitated with ethanol and re-suspended in RNase free water. The RNA concentration was measured with a Qubit Fluorimeter (Thermo Fisher Scientific, Waltham, MA, USA) and the RNA integrity determined using an Agilent 4200 TapeStation (Agilent Technologies, Santa Clara, CA, USA). 500 ng of total RNA from each sample were used for preparing libraries for sequencing, while using an Illumina TruSeq Stranded mRNA HT kit (Illumina, Cambridge, UK), according to the manufacturer’s instructions. Briefly, polyadenylated RNA molecules were captured, followed by fragmentation. RNA fragments were reverse transcribed and then converted to dsDNA, end repaired, A-tailed, ligated to dual indexed adaptors, and PCR amplified. The libraries were pooled in equimolar concentrations and then sequenced in an Illumina NextSeq 500 sequencer (Illumina, Cambridge, UK) using a high output cartridge, generating approximately 44 million single reads, with a length of 75 base pairs (bp). At least 94.8% of the reads generated presented a Q score of 30 or above. 

RNA-Seq reads were quality assessed (FastQC; http://www.bioinformatics.babraham.ac.uk/projects/fastqc) and the sequence adaptors were removed (TrimGalore; https://www.bioinformatics.babraham.ac.uk/projects/trim_galore/).

RNA-Seq reads were aligned to the *Homo sapiens* genome (GRCh38) downloaded via Ensembl while using HISAT2. HISAT2 is a fast and sensitive splice aware mapper, which aligns RNA sequencing reads to mammalian-sized genomes using the FM index strategy [29]. FeatureCount [30] was used to count reads mapping to genes’ annotation files. The read counts were normalized to counts per million (CPM), unless otherwise stated. The edgeR package was used in order to calculate the gene expression level and analyze differentially expressed genes between sample groups [31]. The RNA sequencing data were submitted to European Nucleotide Archive under accession number PRJEB41624. 

Pathway analysis of the differentially expressed genes (absolute FC > 2) was performed while using the R package clusterProfiler [32] with org.Hs.eg.db used for annotation. Enriched GO pathways were identified as GO terms that had a *p*-ajusted value <0.05 after Benjamini–Hochberg correction for multiple testing. One representative term was selected from redundant terms for a similarity >0.5 using the simplify function in clusterprofiler.

The networks of interacting genes up-regulated in PUUV infected A549 cells at day 5 post infection were visualized while using STRING software [33].

### 2.9. RT-qPCR Analysis of mRNA Involved in the IFN Response

The cells were seeded in 12-well plates at 5 × 10^4^ cells/well. Next day, supernatant was removed, and cells were infected at a final MOI of 1 in DMEM 5% FBS. RNA from lysates of infected cells was recovered at different time points using TRI Reagent^®^ (Sigma), following the manufacturer’s instructions, and RNA concentration was quantified with a Nanodrop spectrophotometer (ND1000, Thermo Scientific) and stored at −80 °C for later use. Reverse-transcription was performed using High Capacity cDNA Transcription kit (Applied Biosystems) with random primers. The reactions were carried out in a thermocycler as follows: 10 min at 25 °C, 2 h at 37 °C, 5 min at 85 °C. Quantification of cDNAs was then performed by quantitative PCR using SYBR Green technology (EurobioGreen^®^ Mix qPCR 2X Lo-Rox, Eurobio) with gene-specific primers. The primers for amplifying mRNA encoding IFNs or ISGs (Table S1c) were obtained from online website PrimerBank-MGA-PGA (https://pga.mgh.harvard.edu/primerbank/).

### 2.10. Mass Spectrometry Analysis

MS grade acetonitrile (ACN), MS grade H_2_O, and MS grade formic acid (FA) were purchased from ThermoFisher Scientific (Waltham, MA, USA). Sequencing grade trypsin was from Promega (Madison, WI, USA).

#### 2.10.1. Sample Preparation for Liquid Chromatography Coupled to Tandem Mass Spectrometry (LC-MS/MS)

The HEK293T cells were plated at 10^6^ cells in six-well plates, grown for 24 h, and then transfected with 1 μg of pCiNeo/strep empty plasmid or 1 μg pCiNeo/strep plasmid encoding StrepTag-NSs of PUUV or TULV. Each condition was set up in triplicate and cell lysates were prepared 24 h post transfection in NET/1% TX100 buffer. Pull-down of complexes was performed by incubating the amount of cell lysate corresponding to 1 mg of proteins with 25 µL of packed beads of Strep-Tactin^®^ sepharose (IBA) for 1 h under rotation at 4 °C. After incubation, the beads were washed three times with 1 mL of NET/1% TX100 buffer before a final wash in 25 mM NH_4_HCO_3_ (Sigma).

The beads were incubated overnight at 37 °C with 20 μL of 25 mM NH_4_HCO_3_ buffer containing 0.2 μg sequencing-grade trypsin. The resulting peptides were loaded and desalted on evotips provided by Evosep (Odense, Denmark), according to the manufacturer’s procedure. The samples were analyzed on an Orbitrap Fusion mass spectrometer (ThermoFisher Scientific, Waltham, MA, USA) coupled with an Evosep one system operating with the 30SPD method that was developed by the manufacturer. Briefly, the method is based on a 44-min gradient and a total cycle time of 48 min with a C18 analytical column (0.15 × 150 mm, 1.9 µm beads, ref EV-1106) that was equilibrated at RT and operated at a flow rate of 500 nL/min. The gradient is obtained by flow injection of solvent A (H_2_O/0.1% FA) and solvent B (ACN/0.1% FA). The mass spectrometer was operated by data-dependent MS/MS mode. Peptide masses were analyzed in the Orbitrap cell in full ion scan mode, at a resolution of 120,000, a mass range of *m*/*z* 350–1550 and an AGC target of 4 × 10^5^. MS/MS were performed in the top speed 3 s mode. The peptides were selected for fragmentation by higher-energy C-trap dissociation (HCD) with a normalized collisional energy of 27% and dynamic exclusion of 60 s. Fragment masses were measured in an ion trap in rapid mode, with an AGC target of 10^4^. Monocharged peptides and unassigned charge states were excluded from the MS/MS acquisition. The maximum ion accumulation times were set to 100 ms for MS and 35 ms for MS/MS acquisitions, respectively. 

#### 2.10.2. Quantification of Protein Abundance Variations

Label-free quantitation was performed while using the Progenesis-Qi for proteomics software version 4.2 (Waters, Milford, MA, USA). This software was allowed to automatically align data to a common reference chromatogram to minimize missing values. Subsequently, the default peak-picking settings were used to detect features in the raw MS files and the most suitable reference was chosen by the software for the normalization of data following the normalization to all proteins’ method. In this study, average alignment score and vector numbers were of 97% and 1053, respectively. The normalization factors ranged between 0.82 and 1.29. A between-subject experiment design was chosen to create groups of three biological replicates. The MS/MS spectra were exported and searched against a Uniprot human reference proteome FASTA file modified to include PUUV, TULV, and PHV viral sequences (release 2019, 74,374 entries) while using Proteome Discoverer 2.4 software (ThermoFisher Scientific, Waltham, MA, USA) and the associated SEQUEST software package. A maximum of two missed cleavage sites was authorized. Precursor and fragment mass tolerances were set to, respectively, 7 ppm and 0.5 Da. The following post-translational modifications (PTMs) were authorized as variable PTMs: oxidation (M), phosphorylation (S/T/Y), and acetylation (K/N-terminal). Peptide spectrum-matches (PSMs) were filtered while using a 1% FDR (false discovery rate) threshold calculated with the Percolator algorithm. The identification results were then imported into Progenesis to convert peptide-based data to protein expression data using the Hi-3 based protein quantification method. The data were then processed using multivariate statistics to evidence differentially enriched proteins meeting the following criteria: at least two unique peptides, fold change higher than 2, ANOVA *p*-values lower than 0.05, and power higher than 0.8. Proteins were considered as potential partners of viral proteins if they were enriched in StrepTactin pull-down samples of PUUV, TULV, and PHV NSs proteins, as compared to empty plasmid samples that were used as control. Pathway analysis of the differentially enriched proteins was performed using the R package clusterProfiler with the same parameters as the ones that were used for transcriptomic analysis.

## 3. Results

### 3.1. N, NSs and Glycoproteins of PUUV, TULV and PHV Differentially Inhibit RIG-I-Mediated IFNβ Promoter Activation

**Figure 1:**
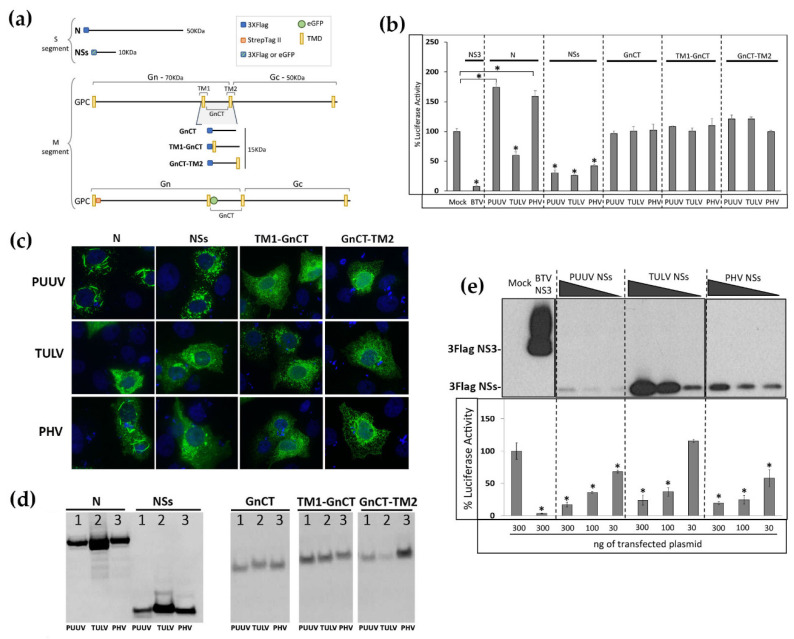
Effect of different hantaviral proteins on RIG-I-induced interferon promoter activity. (**a**) Schematic representation of the different tagged sequences used in our study: 3xFlag-tag (blue square), StrepTagII (orange square), and eGFP tag (green circle) were added to N, nonstructural (NSs) or the different forms of GnCT (GnCT, TM1-GnCT, GnCT-TM2) of pathogenic Puumala virus (PUUV), low-pathogenic Tula (TULV), and non-pathogenic Prospect Hill (PHV). In the case of full-length glycoprotein precursor (GPC), StrepTagII was added in N-ter and eGFP between the TM1 and cytosolic domains of Gn. (**b**) HEK293T cells were transfected with 300 ng of plasmids encoding N, NSs or GnCT variants of PUUV, TULV or PHV, together with the plasmid mix to express RIG-I, along with the IFNβ-Luc and CMV-RL reporter genes. The histogram represents the percentage of luciferase activity 24 h post transfection using mock condition as 100% of IFNβ-Luc activity induced by RIG-I and NS3 of BTV as control of inhibition. (**c**) Immunofluorescence staining of the different 3xFlag-tagged viral proteins was performed in VeroE6 cells, 24 h post-transfection, using an anti–Flag mAb then an anti-mouse IgG coupled to Alexa-488. The images obtained using an objective x63 are at the same magnification (**d**) Protein expression levels of N, NSs, TM1-GnCT, and GnCT-TM2 was controlled by western blot staining with anti-Flag-HRP mAb using 5 μg of lysates from transfected HEK293T. In (**e**), the inhibitory effect of different amount of NSs proteins was tested on RIG-I-induced IFNβ promoter-driven Luc activity (lower panel) and protein expression in the corresponding cell lysates was tested by western blot (upper panel). The results in (**b**,**e**) were obtained from technical triplicates of at least three independent experiments. The error bars correspond to standard deviation to the mean. Statistically representative differences are indicated: * = *p* value < 0.05.

As other negative stranded RNA viruses, orthohantaviruses are recognized by RIG-I/MDA5 receptors triggering the production of IFN [34]. Thus, many viruses inhibit this antiviral pathway in order to be able to replicate [35]. We first evaluated which one of the proteins of pathogenic wild type PUUV Sotkamo strain and low or non-pathogenic TULV Moravia strain and PHV strain could inhibit IFN response, by comparing their effect on human IFNβ promoter activity. We used a luciferase reporter assay that was developed in HEK293T cells transfected with plasmid constructs expressing viral proteins N, NSs, or the cytosolic tails of Gn under different forms allowing for intracellular membrane insertion and cytosolic localization, which could lead to different topological organization, i.e., GnCT, TM1-GnCT, and GnCT-TM2 all fused in frame with a 3xFlag tag at their N-ter (Figure 1a). The nonstructural NS3 protein from Bluetongue virus (BTV), a powerful inhibitor of the IFN activity induced by RIG-I, was used as control [36]. While BTV NS3 was reducing the IFNβ promoter-driven luciferase activity by 94%, no inhibition was observed with the N proteins of PUUV or PHV, and the N of TULV slightly diminished this activity by about 35%. Upon the maturation of GPC into Gn and Gc, the cytosolic tail of Gn (GnCT) faces the cytosolic side of the endoplasmic reticulum (ER) membrane and, since it has been shown that transfected GnCT construct from New York virus (NYV, a SNV strain), ANDV, and TULV can inhibit the IFN response [37], it was of interest to test whether GnCT of PUUV, TULV, and PHV in the different conformations could impact IFNβ promoter activation. However, none of the different GnCT constructs of the three orthohantaviruses were able to antagonize IFNβ promoter-driven Luc activation. In contrast, NSs proteins of all three orthohantaviruses showed a substantial inhibition (60 to 75% reduction) of this IFNβ-Luc activity (Figure 1b).

The cytoplasmic distribution of the Flag-tagged N, NSs, TM1-GnCT, and GnCT-TM2 proteins was then controlled by immunofluorescence staining with anti-Flag antibody, 48 h post-transfection of VeroE6 cells with the different expression plasmids (Figure 1c). The PUUV and PHV N proteins showed a tendency to form filaments in the cytoplasm. The NSs proteins of PUUV, TULV, and PHV appeared to be more aggregated in the cytoplasm with different virus-dependent distribution patterns. Concerning TM1-GnCT and GnCT-TM2, their distribution in the cytoplasm formed membrane networks that are reminiscent of ER organization.

The level of expression of the different viral proteins in lysates of HEK293T cells, which were transfected with the expression plasmids encoding the different Flag-tagged N, NSs, and GnCT proteins, were also tested 24 h after transfection by western blot (Figure 1d). N proteins migrated with an apparent molecular mass of 50 kDa, NSs proteins around 15 kDa and GnCT around 20 kDa. We reproducibly observed higher detection levels for the N of TULV as compared to the N of PUUV and PHV. This is also the case for the NSs of TULV. In contrast, the expression of GnCT was low, either due to a weak expression or a lack of stability and degradation of this domain outside of its natural Gn/Gc assembly context. Of note, only a few cells were efficiently expressing GnCT upon the transfection of the corresponding plasmids.

Because of the differences that were observed in the expression level of viral proteins, we next tested whether the inhibitory effects of NSs proteins of all three viruses on the IFN promoter-driven luciferase activity was dose-dependent. Interestingly, despite its low quantity detected in western blot when compared to TULV NSs (Figure 1e, upper panel), PUUV NSs protein yielded a comparable level of inhibition of luciferase activity as the NSs proteins of TULV and PHV (Figure 1e, lower panel). This might indicate that the pathogenic PUUV NSs had a higher antagonistic capacity on the pathway than low or non-pathogenic orthohantaviruses. Alternatively, the detection of PUUV NSs protein could be reduced due to protease degradation at the site of the Flag-Tag. 

### 3.2. The NSs Proteins of PUUV, TULV and PHV, and the N of TULV, Target Different Steps of the IFN Signaling Cascade

**Figure 2:**
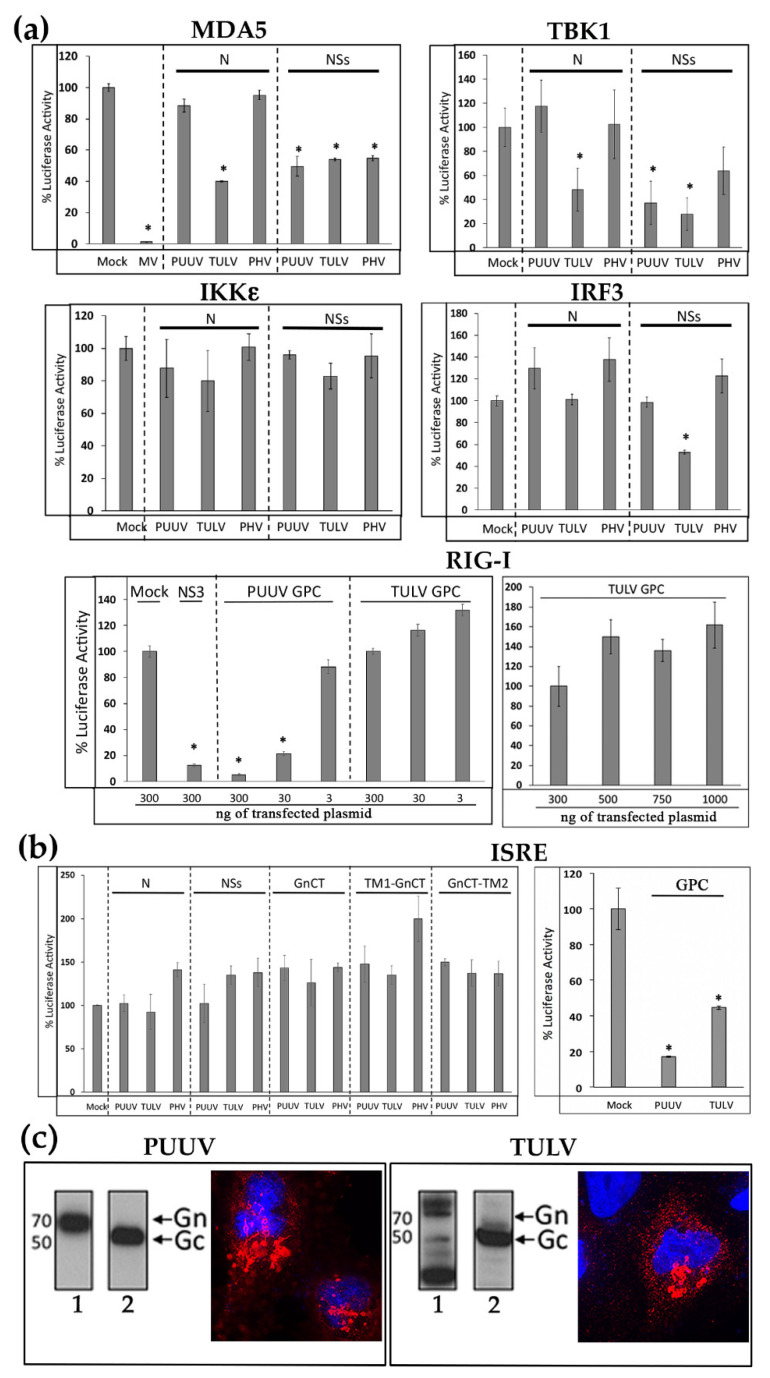
Effect of hantaviral proteins on different elements of the IFN signaling cascade. HEK293T cells were used 24h post-transfection with 300 ng of plasmid encoding 3xFlag-tagged proteins of PUUV, TULV, or PHV, as indicated in the different panels. In (**a**) is shown the inhibitory activity of the different N and NSs proteins on IFNβ Luc activity induced by different elements of the signaling cascade, i.e., MDA5, TBK1, IKKε, or IRF3-5D and of different amount of full-length GPC on RIG-I induced IFNβ-Luc activity. The V protein of measles virus (MV) and BTV-NS3 proteins served as positive control for inhibition of MDA5 and RIG-I-mediated IFN activation, respectively. In (**b**) is shown the effect of plasmid encoding N, NSs, and the different forms of GnCT (left panel) and of GPC (right panel) on ISRE-Luc activity triggered by adding 1000 IU of human IFNβ to transfected HEK293T cells. The histograms represent the percentage of firefly luciferase activity obtained from three independent experiments performed in triplicates, error bars correspond to standard deviation to the mean and asterisk to statistically significant differences with a *p* value < 0.005. (**c**) The correct maturation of Gn and Gc glycoproteins from PUUV and TULV GPC constructs transfected in VeroE6 cells was confirmed by western blot assay. Gn (lane 1) was detected using anti-StrepTag-HRP mAb and Gc (lane 2) while using the mAb 10B8 and then anti-mouse IgG coupled to HRP. Immunofluorescence staining of Gn in red was obtained using anti-StrepTag mAb and then anti-mouse IgG coupled to Alexa 555. The nuclei were stained in blue with DAPI.

The N protein of TULV and NSs proteins of PUUV, TULV, and PHV, which were found to inhibit IFN promoter activation by RIG-I, were further tested by luciferase reporter assays in order to determine the downstream elements of the IFN signaling pathway with which they could interfere. We expressed either MDA5, acting at the same level as RIG-I, TBK1, or its molecular counterpart IKKε (both activated by MAVS), or IRF3-5D, in order to activate the IFN promoter at different levels (Figure 2a). As expected, no inhibition of the luciferase activity was detected in HEK293T cells co-transfected with 3xFlag-N constructs of PUUV or PHV. In contrast, the 3xFlag-N construct of TULV inhibited TBK1 albeit neither IKKε, nor IRF3 induced IFN promoter activation, which suggested that the TULV N protein probably operated upstream of IRF3. Similar observations were made with 3xFlag-NSs protein of PUUV, as it inhibited the TBK1-activated pathway upstream of the IRF3 step, while 3xFlag-NSs of TULV inhibited the pathway at the IRF3 level (or downstream) of the cascade, although only weakly reducing the IFNβ-Luc activity by 40%. This suggested that these two NSs proteins could act on different signaling elements. The NSs protein of PHV, similarly to that of PUUV, could act upstream of IRF3. Again, none of the NSs proteins inhibited the pathway at the level of IKKε.

Some of bunyaviral NSs proteins have been shown to inhibit both the activation of IFN production and downstream amplification of the JAK/STAT pathway activated by IFN, leading to activation of many ISG [17]. In order to evaluate this possibility, the HEK293T cells were co-transfected with plasmids encoding viral proteins and ISRE reporter constructs. We did not observe any inhibitory effect of N, NSs and the different forms of GnCT protein domains on the ISRE promoter-driven Luc activation induced by human recombinant IFNβ (Figure 2b).

### 3.3. The Glycoproteins of PUUV, but Not TULV, Inhibit RIG-I-Induced IFNβ Activation, While Both of Them Interfere with ISRE Promoter Activation

It has been proposed that the cytosolic tail of Gn could have a regulatory role through interaction with cellular factors of the innate immunity [37,38]. While different constructs expressing the cytosolic tail of Gn were not able to inhibit the IFNβ promoter activity (Figure 1b), PUUV GPC gave rise to a strong and dose dependent reduction of the IFNβ-Luc activity that was triggered by RIG-I (Figure 2a). In contrast, TULV GPC had no effect, even when the dose of plasmid was increased to 1 μg (Figure 2a, RIG-I right panel). When the effect of these viral glycoproteins was tested on the JAK/STAT pathway activated by recombinant IFNβ, the GPCs of both PUUV and TULV showed an inhibitory effect on ISRE-Luc activity (Figure 2b, ISRE, right panel). 

Of note, this protein migrated, as expected, with a 50 kDa apparent molecular mass, indicating that maturation of PUUV GPC or TULV GPC occurred correctly 24 h post transfection in HEK293T cells, as shown in western blot using an antibody detecting Gc (Figure 2c, lane 2). However, differences were observed in the migration of PUUV Gn as compared to TULV Gn using anti-StrepTag antibody (Figure 2c, lane 1). While one band of 70 kDa corresponding to the expected glycosylated form of Gn was observed for PUUV, two bands around 70 kDa were visible in the case of TULV Gn, which suggested a different level of glycosylation as well as a smaller band that could arise from degradation. This could be due to the high level of expression that was observed for TULV proteins. Therefore, we also assessed by immunofluorescence the localization of Gn proteins using anti-StrepTag antibody (Figure 2c). We found both Gn from PUUV and TULV associated to membrane structures close to the nucleus, typical of the distribution of Gn in infected cells, which co-localized with the Golgi apparatus (Figure S1).

### 3.4. Combination of Hantaviral Proteins Differently Inhibit RIG-I-Mediated IFNβ Promoter Activation

**Figure 3:**
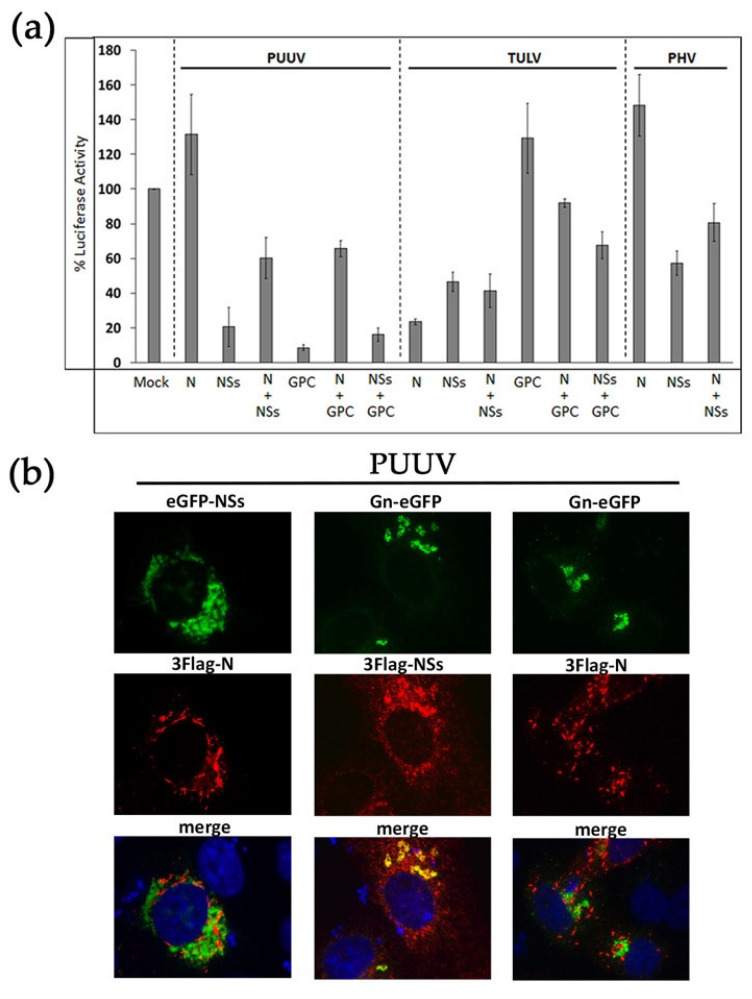
Effect of co-expressing different hantaviral proteins on RIG-I-induced IFN promoter activity and subcellular localization in transfected cells. (**a**) HEK293T cells were transfected with 300 ng of plasmid encoding either 3×Flag-N, 3×Flag-NSs, or StrepTag-GPC individually or in combination (N+NSs, N+GPC or NSs+GPC) using 150 ng of each plasmid. Inhbitory effect of the constructs was measured on RIG-I-induced IFNβ-Luc activity, as described in Figure 1. In (**b**) such combinations of PUUV constructs were co-transfected in VeroE6 cells. Intracellular localization of N with eGFP-NSs (left panels), and of Gn-eGFP with NSs (middle panels) or with N (right panels) were visualized by immunofluorescence. Proteins directly coupled to eGFP appeared in green, and N or NSs tagged proteins stained with anti-Flag mAb, then anti IgG-Alexa 555, appeared in red. The nuclei were labeled in blue with DAPI. The merge panels are shown at the bottom of the figures and co-localizing proteins appearing in yellow. The images obtained using an objective x63 are at the same magnification.

Our comparative study highlighted that the inhibitory effects that were observed on IFNβ promoter-driven luciferase activity depend both on the type of viral protein and the orthohantavirus species. Therefore, we next tested the effect of combining the different proteins of the same virus on the IFNβ-Luc activity that was induced by RIG-I in order to evaluate their overall contribution. Whatever the tested mixture of viral proteins, we observed that the resulting inhibition was always an average of the effect of each individual viral protein, both for PUUV and TULV (Figure 3a). 

In the case of PUUV, while the N protein did not inhibit the IFN pathway and NSs protein reduced the IFNβ-Luc activity to 20%, a mix of the two proteins led to 60% of luciferase activity, which suggested that N weakened the effect of NSs protein. The same was observed when mixing the N and the GPC constructs of PUUV. In this case, the IFN promoter-driven activity raised 70%, despite the low level of IFN activity detected following the transfection of PUUV GPC construct alone (less than 10%). Interestingly, when mixing GPC and NSs constructs, they both efficiently antagonized IFN signaling and the resulting IFN activity was reduced to 17%. We also looked at the cellular localization of co-transfected PUUV constructs (Figure 3b). While N and NSs proteins appeared in different cytoplasmic regions (left panels), it is noticeable that, concomitant to the high inhibitory effect observed with a mix of PUUV NSs and PUUV GPC constructs on IFNβ promoter-driven Luc activity, these two proteins partly co-localized at the level of the Golgi, nearby the viral glycoproteins (middle panels). In contrast, although it has been shown that the N protein and GPC of orthohantaviruses could interact during particle assembly [39], they did not co-localize in transfected VeroE6 cells (right panels). 

In the case of TULV, N and NSs proteins expressed alone had an inhibitory effect reducing the IFNβ promoter activity to 25% and 50%, respectively, and the co-expression of these two proteins still resulted in inhibition being an average (40%). TULV GPC did not antagonize this pathway and, in the presence of TULV N or NSs proteins, inhibition was kept low (Figure 3a). The same was observed after the co-transfection of N and NSs from PHV, when only NSs protein displayed an inhibitory activity.

### 3.5. NSs Proteins Have Different Sequence Characteristics: A NoLS Sequence is Present in TULV NSs

#### 3.5.1. NSs Sequence Analysis 

**Figure 4:**
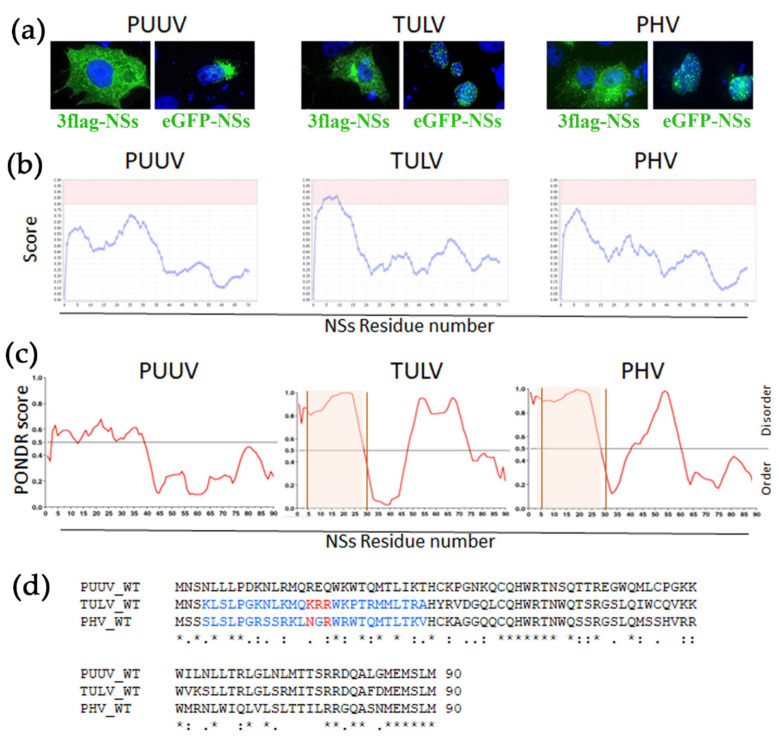
Identification of nucleolar like sequence (NoLS) in NSs proteins. (**a**) Subcellular localization of 3xFlag-NSs (left panels) and eGFP-NSs (right panels) of PUUV, TULV and PHV was obtained by immunofluorescence performed in VeroE6 cells at day 2 post transfection. 3xFlag-NSs was detected with anti-Flag mAb, then anti-mouse IgG Alexa-488 antibody, while eGFP-NSs was directly fluorescent. (**b**) shows predicted NoLS of NSs proteins of PUUV, TULV, and PHV determined by NoD software. Scores higher than 0.8 are highlighted in pink. (**c**) Prediction of naturally disordered NSs regions of the three orthohantaviruses was obtained by in silico analysis with the PONDR^®^ software and NoLS are highlighted in orange. (**d**) shows alignment of amino acid sequences of NSs from PUUV, TULV, and PHV, along with conserved amino acid residues (dots and stars below the set of sequences). The predicted NoLS sequence of TULV and the hypothetical one for PHV are highlighted in blue and the conserved polar residues in red.

Surprising observations were made while testing the localization of hantaviral proteins of TULV in transfected VeroE6 cells. Flag-tagged N (Figure 1c) or NSs (Figure 1c and Figure 4a, left panels) of PUUV, TULV, or PHV all appeared in the cytoplasm, albeit exhibiting different aspects. When an eGFP fluorescent tag was added in N-ter of PUUV NSs protein, it also gave rise to its cytoplasmic localization, but, in the case of TULV, and, to a lesser extent, of PHV, it led to nuclear localization of the NSs (Figure 4a, right panels). 

The same was observed when the fluorescent tag was added in C-ter of TULV NSs, or by using another tag, mCherry (Figure S2). This prompted us to search for physical features in the amino acid sequence of the different NSs proteins. A nucleolar like sequence (NoLS) has been found in the NSs of Schmallenberg virus, another bunyavirus [40]. Thus, we performed an in silico prediction analysis and found a putative NoLS sequence in TULV NSs protein (aa 4–29), which was not found in PUUV NSs. In the case of PHV NSs, its amino acid composition in N-ter appeared close to the threshold of a predicted NoLS (Figure 4b). In addition, the analysis of the NSs amino acid sequences with the PONDR software (Figure 4c) showed that the NSs of low and non-pathogenic viruses, TULV, and PHV displayed a higher level of disordering than PUUV-NSs protein in the N-ter region containing the NoLS. Of note, protein disordering is a feature that is usually associated to a high capacity of interaction [41,42]. The NoLS corresponding regions of TULV and PHV possess a polar motif positively charged, KRR_16–18_ and NGR_16–18_ respectively, while, in the PUUV NSs protein, different polar residues with positive and negative charges were found in the corresponding region, REQ_16–18_, as shown by alignment of the NSs protein sequences (Figure 4d). 

#### 3.5.2. Validation of a NoLS Motif and Its Role in Nuclear Localization by Site-Directed Mutagenesis

**Figure 5:**
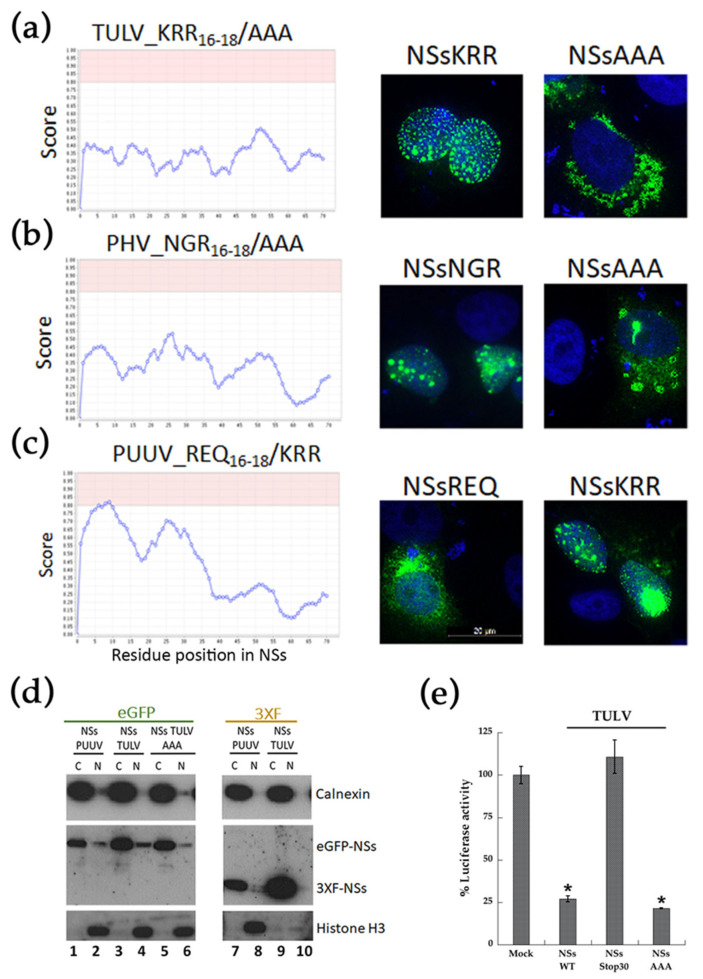
Role of NoLS in perinuclear localization of TULV, PHV and PUUV eGFP-NSs. The polar residues KRR, or NGR at positions 16–18 of TULV and PHV NSs proteins, were replaced with neutral amino acids AAA by site-directed mutagenesis. In silico analysis of an absence of NoLS motif in the engineered NSs AAA_16–18_ sequences is shown in the left panel of (**a**) for TULV and (**b**) for PHV. The cellular localization in VeroE6 cells transfected with 500 ng of the corresponding plasmids encoding eGFP-NSS AAA_16–18_, as compared to the wild type counterpart KRR_16–18_ for TULV and NGR_16–18_ for PHV, is shown in the right panels of (**a**,**b**), respectively. In (**c**) is shown a reverse approach introducing KRR residues at amino acid positions 16–18 of PUUV NSs. Both, in silico analysis (left panel) and immunofluorescence staining of VeroE6 cells (right panel), transfected with PUUV eGFP-NSs KRR_16–18_ construct, were performed. The nuclei were stained in blue with DAPI and the different eGFP NSs proteins emitted green fluorescence. In (**d**) VeroE6 cells transfected with 3 µg of plasmid constructs encoding the eGFP- or 3xFlag-NSs of PUUV or TULV with wild type or mutated NSs AAA_16–18_ sequence were tested by immunoblotting as indicated. Cytoplasmic (C) and nuclear (N) fraction enrichment was determined by the imunodetection of specific markers: calnexin for the cytoplasmic compartment and histone H3 for the nuclear compartment, while eGFP-NSs or 3xFlag-NSs were detected using tag specific antibodies. In (**e**) the different TULV NSs variants (WT, NSsStop30 or AAA_16–18_) were tested for their capacity to inhibit the IFNβ-Luc activity induced by RIG-I. Statistics were performed on replicates of at least three independent experiments and data represent mean +/− standard deviation to the mean (* = *p* < 0.05).

Site-directed mutagenesis evaluated the possible involvement of the polar NoLS motif in the localization of TULV eGFP-NSs at the nucleus. When non-polar alanine residues (AAA_16–18_) replaced the polar motif of TULV NSs (KRR_16–18_), the score analysis for the mutant NSs protein no longer predicted a NoLS (Figure 5a, left panel) and, as shown by immunofluorescence eGFP-NSs (AAA_16–18_), then redistributed to the cytoplasm (Figure 5a, right panel). The same was observed by replacing the NGR motif of PHV NSs by three alanine residues (Figure 5b). This NSs characteristic, depending on the presence of a positively charged motif, was further confirmed by interchanging the negative polar motif REQ in PUUV NSs sequence with this KRR motif (Figure 5c, left panel). Interestingly, PUUV NSs KRR then formed big aggregates that were associated to the nucleus, as shown by fluorescence microscopy (Figure 5c, right panel). 

Our study suggested that the large dots of TULV NSs protein could be located around the nucleus, as is the case of some proteins interacting with nuclear pores [43]. Therefore, we performed cytoplasmic and nuclear protein fractionation from lysates of VeroE6 cells that were transfected with eGFP-NSs or 3xFlag-NSs proteins (Figure 5d). Western blot analysis indicated that NSs majorly partitioned with the cytoplasmic fraction. As expected, histone H3 was associated to the nuclear fraction (Figure 5d, lanes 2, 4, 6, 8, 10) and calnexin, an ER resident protein, to the cytoplasmic one (Figure 5d, lanes 1, 3, 5, 7, 9). Indeed, TULV eGFP-NSs protein was enriched in the cytosolic fraction (lane 3 vs. 4), as is the case for PUUV eGFP-NSs (lane 1 vs. 2) and the eGFP-NSs AAA mutant protein of TULV (lane 5 vs. 6). These results confirmed that, although it was found to localize at the nucleus by immunofluorescence, the TULV eGFP-NSs protein was partitioned with cytoplasmic fraction, rather than with nuclear fraction, similarly to the 3xFlag-NSs protein of TULV (lane 9 vs. 10) and PUUV (lane 7 vs. 8), otherwise localizing in the cytoplasm. A perinuclear rather than nuclear localization of TULV eGFP-NSs was also supported by fluorescence confocal analysis of VeroE6 cells that were transfected with TULV eGFP-NSs construct and co-stained with anti-lamin antibody detecting this nuclear membrane protein (Figure S2b). Besides, the AAA amino acid exchange introduced into TULV NSs protein did not knock down the inhibitory effect of the protein on RIG-I-induced IFNβ-Luc activity. In contrast, the introduction of a stop codon after the NoLS motif of TULV NSs, at codon position His30, abolished the capacity of TULV NSs protein aa 1–29 to inhibit IFN promoter-driven Luc activity (Figure 5e). This could be linked to the fully nuclear localization of TULV NSsStop30 (Figure S2b), impairing its cytoplasmic function, or to the absence of part of the NSs protein downstream of the NoLS, which could carry the IFN promoter inhibitory function. 

### 3.6. A Complex Role of NSs Proteins from PUUV and TULV on IFN Response in Infected Human Cells 

Our luciferase reporter assay demonstrated the functional capacity of all three orthohantavirus NSs proteins to inhibit IFN promoter activity. Besides, NSs amino acid sequence analysis showed particular features that are associated to virus species, which could lead to different ways to interact with cellular factors. Finally, other viral proteins could contribute to the regulation of the IFN response by a particular orthohantavirus. We next evaluated the role of NSs proteins from different orthohantaviruses in infectious contexts using mutant viruses encoding truncated NSs proteins. The PUUVΔNS viral clone, as derived from PUUV Sotkamo, possesses a stop codon that is located 21 codons downstream of the initiating methionine of NSs protein, which did not affect the N protein. It was compared to the PUUV Sotkamo parental strain expressing the full-length NSs, 90 aa long. We also studied two TULV strains: Tula Lodz with a full-length NSs and a variant of Tula Moravia, which has spontaneously acquired a mutation during cell culture passages, thereby introducing a stop codon at codon position 15 of its NSs sequence [23] without affecting the TULV N protein.

#### 3.6.1. Impact of PUUV and TULV NSs Expression on Viral Replication and IFN Induction in A549 Cells 

**Figure 6:**
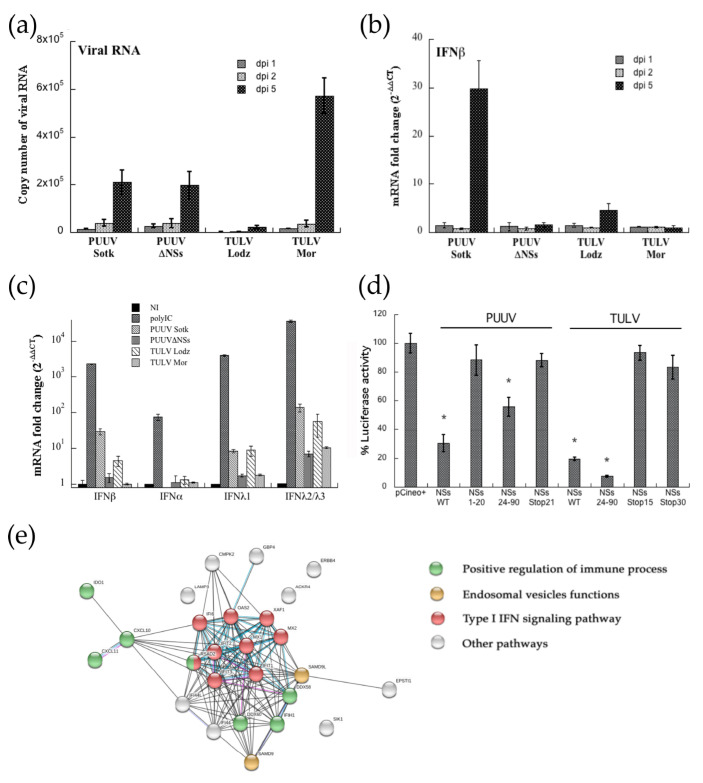
IFN production and networks of interacting genes up-regulated in infected A549 cells. A549 cells, were infected at MOI 0.5 with PUUV or TULV, and their variant forms, defective in expression of a full-length NSs. The histograms correspond to RNA quantified by RT-qPCR and error bars correspond to standard deviation obtained from three independent experiments, evaluating, in (**a**), the number of viral RNA copies in infected A549 cells at dpi 1, 2 and 5, in (**b**), the log2 fold change level as compared to non-infected cells of IFNβ mRNA in the same extracts and in (**c**), mRNA expression levels of IFNβ, IFNα or INFλ1 or IFNλ2/3 at dpi 5 as compared to A549 cells, non-infected, or treated with 1 μg of poly-IC. In (**d**), mutated NSs constructs with a stop codon in position 21 of PUUV NSs or 15 of TULV NSs as well as NSs fragments covering N-ter or C-ter regions were tested for their capacity to inhibit IFN-Luc activated via RIG-I. Standard deviation and statistics (* = *p* < 0.05) were obtained from biological triplicates. In (**e**), up-regulated genes that were identified by RNAseq in PUUV infected A549 cells were analyzed while using STRING software. The edges of known interactions in the network appear in light blue for curated data and in pink for experimentally determined interactions, while black lines correspond to interaction of correlated genes and violet to genes sharing orthology. Relevant nodes are colored, as indicated in the legend.

A549 cells were infected at MOI 0.5 and tested by RT-qPCR at different days post-infection. Replication was low, but similar in PUUV or PUUVΔNSs infected cells, and the number of viral RNA copies of the S segment increased from day 2 to day 5 in RNA extracted from infected cells, as shown in Figure 6a. A higher number of viral RNA copies was detected for TULV Moravia, while a low level of replication was observed with TULV Lodz. The same RNA samples were used in order to quantify the expression of IFN transcripts. Surprisingly, only PUUV Sotkamo expressing the full-length NSs induced a significant level of IFNβ (Figure 6b). It is to be noticed that this up-regulation, around 30-fold change, as compared to non-infected cells only appeared at day 5 post-infection. Interestingly, a weak induction was also observed with TULV Lodz (five-fold), but not with PUUV or TULV expressing truncated NSs. 

However, when considering the low level of IFNβ induced in A549 infected cells, we tested the expression of IFN-I and IFN-III upon one day of treatment with 1 μg of poly-IC. Because different IFNα are expressed in human cells, we designed degenerate pan-α primers that covered and could amplify, at the same time, IFNα 1, 2, 4–8, 10, 13, 14, and 21 subtypes. Poly-IC induced a substantial level of IFNα, β, λ1, and λ2/3 mRNA with a 10^2^, 2.3 × 10^3^, 4 × 10^3^, and 3.5 × 10^4^ fold change, respectively, as shown in Figure 6c. We could not detect mRNA specific for the different IFNα amplified by the pan-α primers in A549 cells infected with any of the viruses (Figure 6c). In contrast, expression of the different IFN-λ was upregulated in A549 cells. While, as observed for IFNβ, IFNλ1 was at a background level in A549 cells that were infected by PUUVΔNSs and TULV Moravia variant clones, a weak and comparable (eight-fold change) amount of IFNλ1 mRNA was observed with PUUV Sotkamo and TULV Lodz both having an intact NSs. Interestingly, A549 infected by PUUV Sotkamo WT, but also TULV Lodz, exhibited a high level of IFNλ2 and IFNλ3 subtypes (136- and 55-fold change, respectively), also induced (around 10-fold change) with mutant orthohantavirus, PUUVΔNSs, and TULV Moravia (Figure 6c). 

Because orthohantaviruses carrying a mutated NSs replicated similarly in A549 cells as to those carrying a full-length NSs, we next wonder whether NSs with a stop codon or the NSs C-terminal region corresponding to the fragment suspected to be expressed from position Met24 by leaky scanning (Binder et al., 2020, submitted) could still inhibit IFNβ promoter-driven luciferase activity (Figure 6d). We found that only the C-terminal fragment NSs24–90, and not the N-ter part of NSs, could inhibit the IFN promoter-driven Luc activity. This observation was especially valid for TULV and, to a lesser extent, for PUUV. Because neither full-length NSs protein from TULV with a stop codon at position aa15 or aa30, nor PUUV NSsStop21, could inhibit IFN-Luc activity, no leaky scanning initiating at NSsMet24 seemed to occur in our reporter gene system and it could be the same in infected A549 cells.

#### 3.6.2. Transcriptomic Analysis of Up-Regulated Genes in A549 Cells Infected by PUUV Sotkamo

The observation of a clear induction of IFNβ mRNA in A549 cells that were infected with PUUV Sotkamo wild-type, and not with PUUVΔNSs, led us to study gene regulation in these cells at dpi 5, as compared to non-infected A549 cells, while using RNA-seq based transcriptomic analysis. Twenty-six genes exhibited a statistically significant up-regulation (log2 fold change > 2, *p*-value < 0.05), as shown in Table 1. Half of them related to antiviral responses, consistent with regulation of IFN signaling pathways. The corresponding protein–protein association network generated while using STRING software also supported this (Figure 6e). For instance, we found IFN-I stimulated genes, such as IFI44, 44L, XAF1, GBP4, MX, and IFN-II induced chemokines, such as CXCL10. We also found helicase transcripts with the CARD domain used as receptors of RNA viruses (IFIH1/MDA5, DDX58/RIG-I, DDX60), known to induce IFN response, but also to be regulated by IFN. Interestingly, most of these genes were found to be up-regulated during infection with other bunyaviruses [44].

#### 3.6.3. Quantification of RNA Expression of IFN-Induced Genes in A549 Cells Infected with PUUV and TULV Strains

**Figure 7:**
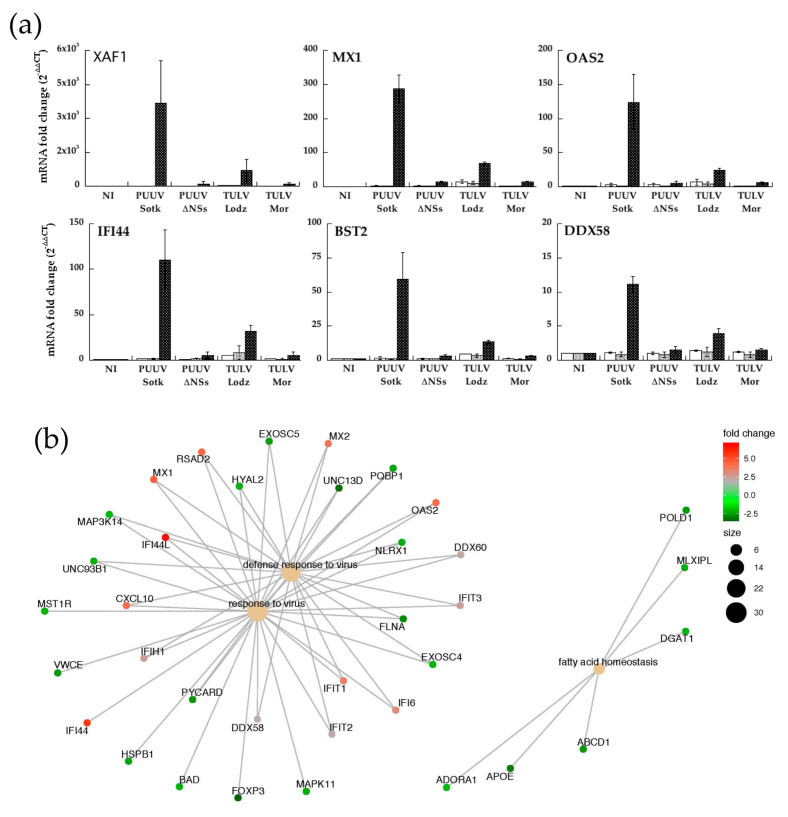
Regulation of expression of genes involved in innate immunity in A549 cells infected by different PUUV or TULV strains. Histograms in (**a**) represent the relative expression (log2 fold change) of XAF1, MX1, OAS2, IFI44, BST2, and DDX58 mRNA determined by quantitative RT-PCR at dpi 1 (white bar), dpi 2 (grey bar) and dpi 5 (black bar) in infected A549 cells. The error bars correspond to standard deviation to the mean of data from biological triplicates. (**b**) Cnetplot visualization of cellular mRNA identified by transcriptomics using R clusterProfiler package implemented in R: central nodes (pale brown) outline over-represented biological functions, and node size corresponds to the number of identified genes associated to them, as shown in the legend. Peripheral nodes represent genes, and their level of regulation, expressed as fold change compared to non-infected cells, displayed using a red-green gradient: strongly up-regulated genes are depicted in red, weakly up-regulated in light grey, and down-regulated ones in green.

mRNA expression of some of the up-regulated genes (XAF1, OAS2, MX1, IF44, RIGI, BST2, DDX58) was tested by RT-qPCR performed on RNA that was extracted from infected A549 cells in order to validate transcriptomic data. The study was enlarged to samples from A549 infected at MOI 0.5 by PUUV and TULV expressing truncated or full-length NSs proteins, as compared to non-infected cells (Figure 7a).

The relative mRNA expression corroborated the results of transcriptomics that were obtained in PUUV infected A549 cells at day 5. Indeed, RT-qPCR showed a high level of expression of XAF1, MX1, IFI44, and OAS2 (>100), while BST2 and RIGI were much less expressed. However, neither PUUVΔNSs nor TULV Moravia, both expressing truncated NSs, induced such gene expression. The genes that were activated by IFN and up-regulated in PUUV infected A549 cells were observed, although at a much lower level, only in TULV Lodz infected cells. These observations correlated well with the fact that no or low IFNβ or IFNλ activation was observed in cells infected with orthohantaviruses expressing truncated NSs. Many genes were down-regulated and those with a fold change |log2| > 2.5 and a *p*-value < 0.0005 are listed in Table S3. 

The main implicated pathways, including up and down regulated genes, were then represented while using cnetplot function of clusterProfiler R package. The investigation revealed that the network of interactions mostly included genes that are involved in anti-viral response, fatty acid homeostasis, and autophagy (Figure 7b).

### 3.7. Cellular Partners in Complex with PUUV or TULV NSs Proteins Were Identified by Mass Spectrometry Analysis

**Figure 8:**
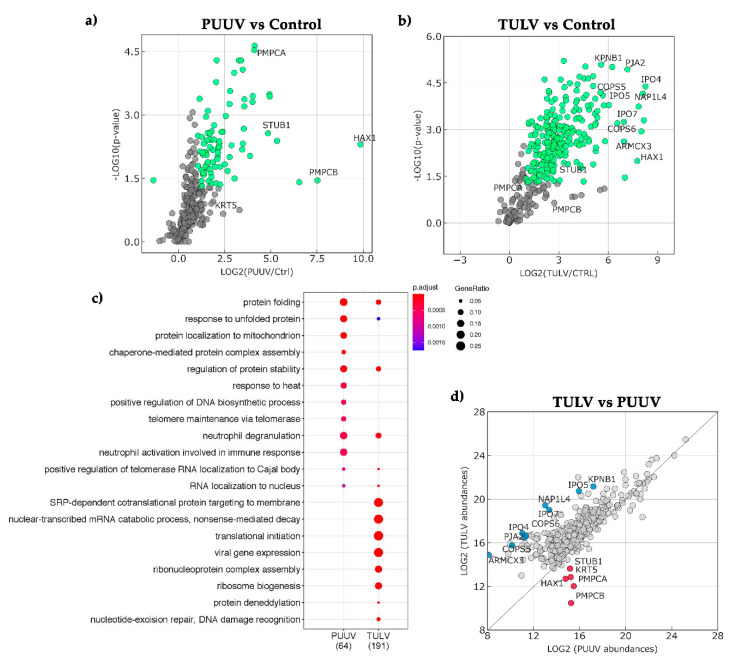
Proteins and their biological processes (BP) associated to pull down of PUUV and TULV NSs complexes. Mass spectrometry analysis was performed on biological triplicates of NSs PUUV and NSs TULV pull-down obtained from cell lysates of transfected HEK293T cells. The identification of human proteins associated to NSs was determined by comparison with the empty plasmid condition. Protein partners and associated BP, were sorted using filters retaining potential partners with *p*-value < 0.05, fold change > 2, more than one peptide found and a power > 0.8. In (**a**), volcano plot of differential pull-down assay of control (Ctrl) *versus* immobilized NSs of PUUV or in (**b**) of TULV are represented. The *X*-axis shows the LOG2 fold change and the *Y*-axis the logarithmic odds of a protein being differentially detected (−LOG10(*p*-value)). Green points stand for sorted proteins, prior to logarithmic transformation, whereas grey points show proteins that do not satisfy fold change and *p*-value criteria. In (**c**), the dot plot visualized the top 20 enriched GO terms of BP that is associated to human proteins specific or common to PUUV and TULV NSs complexes. The number of proteins associated to the NSs proteins of PUUV and TULV, according to the defined criteria, are indicated below the panel. The color of the dots represents the *p*-value adjusted by Benjamini-Hochberg correction for each enriched GO term identified by Fisher’s exact test using enrichGO function in R package clusterProfiler, and the size of the dot represents the number of genes enriched in the total gene set. In (**d**), scatter plot of differential pull-down assay shows the LOG2 of protein abundances associated with immobilized TULV NSs (Y-axis) *versus* immobilized PUUV NSs (X-axis). All plotted values correspond to protein identification with at least 2 unique peptides. Values close to the dotted line correspond to proteins found in similar abundance in the two conditions and the more abundant proteins are indicated for PUUV in red and for TULV in blue.

The differences relating to NSs interactions with antiviral response observed both at a molecular and cellular level led us to compare the cellular proteins that were found in complex with PUUV and TULV NSs proteins. In the absence of antibody to precipitate these proteins in lysates of infected cells, we used the StrepTactin pull-down of lysates of HEK293T cells transfected with plasmid encoding StrepTag-NSs proteins from PUUV or TULV and the pCiNeoStrepTag empty plasmid as background reference. Proteins in complex with StrepTag-NSs were then analyzed by LC-MS/MS and identified from *Homo sapiens* dataset. While biological processes identified with the highest score for partners of PUUV NSs were majorly involved in protein folding and stress defense, they appeared to relate more to RNA and protein metabolism as well as protein targeting in the case of TULV NSs, as shown in Figure 8a. 

Although a smaller number of proteins were precipitated in complex with PUUV NSs as compared to TULV NSs (Figure 8b), at a ratio of 64 versus 191, two proteins associated to mitochondrial function, HAX-1, an antiapoptotic protein and subunit α and β of a mitochondrial processing peptidase involved in mitochondrial import were the most abundant in PUUV NSs protein complexes. STUB1, an E3 ubiquitin protein ligase also appeared more abundant in PUUV than in TULV NSs complexes. Concerning proteins interacting with TULV NSs protein, besides cellular proteins involved in RNA metabolism, transcription and translation, we found, as more abundant proteins, some importin subunits (IPO4, 5, 7 or KPBN1), signalosome components that are involved in endocytosis or hypoxic stress (COP1, 2, 4), proteins associated to mitochondrial function (ARMCX3, NIPSNAP1). Other identified human proteins, such as RNH1, which is an inhibitor of RNAse, ABCD3 found to be associated to replication sites of picornaviruses, and CSNK2A1, which controls cell cycle and apoptosis, are of interest when comparing hantaviral NSs interaction partners.

## 4. Discussion

Partly due to the great diversity of orthohantaviruses and their natural reservoirs, interactions of these viruses with the host immune system leading to persistence in rodents and pathogenicity or tolerance in humans are not fully understood [45]. Moreover, reverse genetics systems are not available and, since these viruses are not pathogenic in rodents, there are no adequate animal models to apprehend mechanisms of their pathogenesis. Different studies have shown that orthohantaviruses can interfere with the IFN response at different levels of the signaling pathway, but no comprehensive picture has emerged yet [46]. In order to better understand the role of orthohantavirus proteins relating to IFN signaling, here we compared the effect of N, NSs, and different forms of the cytosolic domain of Gn proteins, expressed alone or in their GPC context of maturation, for three orthohantaviruses, PUUV, TULV, and PHV. 

Using an IFNβ promoter-driven luciferase reporter assay, here we described different antagonisms of the IFN pathway activated via RIG-I and, to a lesser extent, MDA5, depending on viral proteins and orthohantavirus species. We found an inhibition of the IFNβ promoter by the N of the low-pathogenic TULV and all three NSs proteins from PUUV, TULV, and PHV occurring at the level of TBK1. In contrast, the N of PUUV and PHV did not inhibit, and even increased, the IFN promoter-driven Luc activity. Such an enhancing capacity to stimulate RIG-I-activated antiviral response in A549 cells has also been described for the N of HTNV [47].

Regarding the cytosolic tail of the envelope glycoprotein Gn of PUUV, TULV, or PHV, no inhibition of the IFN promoter activity that was induced by RIG-I was measured with any of the different forms of GnCT used in the present study. This was unexpected for PUUV, in regards of studies showing that GnCT from pathogenic NYV and ANDV, but not from non-pathogenic PHV, inhibit IFN activation via RIG-I by disrupting TRAF3-TBK1 interaction [48,49]. This was attributed to the presence of a degron signal in the cytosolic tails of pathogenic orthohantaviruses [50], but Gn degradation of the low-pathogenic TULV is also described [51], as well as an impact of its GnCT on TBK1 [52]. These discrepancies could be specific to virus strains or plasmid constructs, and also depend on the cellular models that were used in the different studies. In our case, although the different GnCT forms appeared as membrane networks localizing in the cytoplasm of transfected cells, we cannot warrant that GnCT, outside of its Gn/Gc assembly context, could be correctly inserted, in the right orientation, at the cytoplasmic side of ER membranes. Therefore, we transfected full-length GPC constructs in order to express envelope glycoproteins in a more native conformation, which resulted in the expression and cleavage of GPC into Gn and Gc. Interestingly, PUUV GPC, but not TULV GPC, inhibited RIG-I-induced IFN promoter activation, while they both antagonized the JAK/STAT signaling pathway that is induced by human recombinant IFNβ. This was in accordance with the results from the literature describing that glycoproteins from ANDV and PHV inhibit the nuclear translocation of STAT1 induced by IFNβ in transfected VeroE6 cells [12]. We did not observe inhibition of STAT1 phosphorylation (Figure S3) and it would be of interest to evaluate whether the observed antagonistic effects could be due to inhibition of nuclear translocation of transcription factors, through their sequestration by viral proteins [53,54].

The different effects of individual proteins from PUUV, TULV, and PHV on IFNβ- and ISRE promoter-driven luciferase activities highlighted the complexity of interactions that different orthohantaviruses have established during long-term evolution with their hosts. For instance, it has been shown that the N protein of HTNV interacts with importin-α and, in this way, inhibits tumor necrosis factor alpha (TNFα) activated by NFκB. Consistent with the block of NFκB nuclear translocation, the N proteins of Seoul (SEOV) and Dobrava-Belgrade (DOBV) orthohantaviruses also interact with importin-α, while at the same time, the N proteins of PUUV, SNV, and ANDV do not inhibit NFκB [55,56]. It has been shown that the N protein of ANDV, but not of SNV or PHV, inhibits the cascade at the level of TBK1/IKKε [57], but another study shows the inhibition at the level of PKR phosphorylation [58]. In the case of the N protein of Laguna Negra orthohantavirus (LNV) and Maporal orthohantavirus (MAPV), interference with the antiviral response occurs at the level of STAT phosphorylation [59]. These contrasting observations suggest an inhibitory role on IFN signaling, which is virus specific rather than related to the induced disease or its severity. It might reflect evolution of strategies used by these viruses to antagonize antiviral response. When we compared the viruses with each other, we observed that the NSs and GPC of PUUV, but not its N, inhibited the IFNβ promoter-driven activation, while the N and NSs of TULV, but not its GPC, possessed inhibitory activity. To be added, GPC of both PUUV and TULV, but no other viral proteins, inhibited the IFNβ induced ISRE promoter activation. Interestingly, co-transfection that was achieved by mixing viral proteins in the luciferase reporter assay led to additive effects of each contributing protein. The strongest inhibition of IFNβ-Luc activity was obtained by co-transfecting NSs and GPC constructs of PUUV, which surprisingly also co-localized at the level of the Golgi apparatus. Therefore, it suggested that GPC could have a major role in antagonizing IFN response.

It was then tempting to speculate that the different hantaviral proteins could contribute to interaction with the IFN response during infection in a virus dependent manner and that, in such a context, the kinetics of expression of the different proteins during the viral cycle would also be important. Because NSs of all three orthohantaviruses appeared to be efficient antagonists of RIG-I-induced IFN-I activation, we investigated the role of NSs during viral infection by taking advantage of viral strains expressing either a full-length NSs (PUUV Sotkamo wild-type, TULV Lodz) or a truncated NSs (PUUVΔNSs, TULV Moravia). Only a few studies have suggested a role of hantaviral NSs in IFN antiviral response [21]. A recent study described the detection of ANDV NSs in lungs of infected Syrian hamsters used as model of pathogenesis, and it has shown that ANDV NSs antagonizes the IFN-I signaling pathway by disrupting MAVS-TBK1 interaction [60]. It has been published that PUUV deficient for NSs expression was 10 times less infectious in A549 interferon competent cells than the wild-type virus strain [7]. However, we only find little differences when comparing PUUV and PUUVΔNSs viral replication in IFN competent A549 cells (Figure 6a and Binder et al., 2020, submitted). Additionally, in infected A549 cells, the replication of TULV Moravia encoding a truncated NSs [23] was higher than replication of TULV Lodz with full-length NSs. These data did not point out any clear correlation between orthohantavirus replication and NSs-ORF expression.

Although the pre-treatment of cells with different IFN-I and IFN-III subtypes, as well as with IFNγ, has been shown to interfere with orthohantavirus replication, the question remains on the type of human IFN that is induced during orthohantavirus infection. Indeed, in vitro and in vivo, only small amounts of IFN-I and IFN-III are detectable, so that orthohantaviruses could be considered to be weak inducers of IFN [4,9]. Our luciferase reporter assay only related to the IFNβ response, while A549 cells are competent in expressing different IFN-I and IFN-III subtypes. In this cell line, PUUV Sotkamo and TULV Lodz expressing a full-length NSs induced a low but significant level of IFNβ transcripts, (30- and five-fold change, respectively), while variants with truncated NSs did not. Moreover, while none of the four orthohantaviruses induced IFNα in A549 cells, IFNλ1, λ2, and λ3 were significantly induced in these cells infected by PUUV Sotkamo wild type and TULV Lodz and, interestingly, also by the viruses encoding a truncated NSs. In the IFNβ reporter assay, the different truncated forms of PUUV and TULV NSs (residues 1–20 and 1–14, respectively), as well as the mutated full-length NSs-ORF, had no inhibitory effect on IFNβ promoter-driven Luc activity. In contrast, PUUV and TULV NSs fragments corresponding to the C-terminal amino acid residues 24–90, potentially expressed from the Met 24 residue by leaky scanning, were still inhibiting, in particular with high efficiency in the case of TULV NSs 24–90. Therefore, if stop codons abolished NSs activities and if this viral protein was not the only player in inhibiting IFNβ, as supported by the antagonistic role of other hantaviral proteins, then it was consistent with the fact that we did not observe differences in the replication of orthohantaviruses with complete or truncated NSs proteins. However, the same should apply to IFN production, and it was then puzzling that low or no IFN induction was observed, whatever β or λ, with viruses that are deficient in full-length NSs expression, whereas NSs had the highest inhibitory impact in the luciferase assay. 

This led us to ask the question whether NSs, besides its role in IFNβ signaling, as revealed in our reporter assay, could be involved in the regulation of other cellular pathways and whether N-ter or C-ter domains of NSs protein could harbor different roles. While the NSs proteins of Arvicolinae-associated orthohantaviruses are conserved in size and location on the S segment, it is noticeable that, in contrast to their conserved N amino acid sequence, the NSs proteins exhibit divergent sequences, although multiple Met codons are preserved (Binder et al., 2020, submitted). In silico analyses also revealed differences in sequence disorder and presence of a NoLS at the N-ter of TULV NSs protein exhibiting a polar motif (KRR_16–18_). Interestingly, investigation of intracellular NSs localization using different tag and transiently expressed NSs proteins showed that, while NSs proteins from PUUV, TULV, and PHV that were coupled to small tags were found in the cytoplasm of transfected VeroE6 cells, eGFP, or mCherry fluorescent tags added in N-ter or C-ter, led to nuclear routing of TULV NSs, but not of PUUV NSs. The direct role of the polar residues in the nuclear addressing of TULV NSs was demonstrated by inactivation of the motif or by introducing it in NSs sequences of PUUV or PHV using site-directed mutagenesis. Altogether, these data highlighted that the N-ter region of NSs could carry features allowing for interaction with cellular proteins in different ways. Such interactions could then vary according to cell type or species of origin, and they could occur either directly or indirectly and depend on the virus species and strain. The C-terminal region could then be involved in organization and localization of NSs important to achieve its function either as IFN antagonist or as regulator of another pathway albeit linked to IFN production. This question was further approached by two ways: (i) transcriptomic analysis of RNA expression in infected A549 cells and (ii) the identification of partners of NSs proteins expressed by transfection of the corresponding constructs in HEK293T cells. 

Transcriptomic analysis of PUUV infected A549 cells highlighted the induction of genes that are involved in the IFN-induced antiviral response as well as pro-inflammatory cytokines, which could be important for PUUV pathogenesis. Interestingly, in line with IFNβ expression, ISG genes were also activated, although weakly, in cells that were infected with TULV Lodz, but not in cells infected with variant viruses PUUVΔNSs and TULV Moravia. It was also noticeable that many genes appeared as being down regulated. Software analysis of gene ontology or reconstructed pathway revealed that the main pathways, including up and down regulated genes, belong to the innate immune response, including response to viruses and cellular antiviral pathways. Fatty acid homeostasis was also represented in A549 cells that were infected by PUUV, a biological function that has shown to be important for the replication of other bunyaviruses both by exerting an antiviral role [61] or by supporting viroplasm like structure formation [62].

Besides, comparison by LC-MS/MS analysis of cellular proteins that were found in complex with NSs of PUUV or TULV also pointed to possible other roles of NSs proteins. We speculated that the presence of polar residues in the disordered region of NSs could be involved in interactions with different cellular factors. Indeed, the unequivocal enrichment in proteins that are associated with NSs of PUUV and TULV and involved in different biological processes supported this hypothesis. Pathways that are involved in protein folding and unfolded protein response, as well as mitochondrial function, were predominant in PUUV-NSs complexes. Of interest, HAX-1 involved in apoptosis has been described to interact with a nonstructural protein of influenza virus [63] and a mitochondrial peptidase to play a role in assembly of rubella virus capsid [64]. Noticeably, HAX-1, which is enriched in PUUV NSs protein complexes, has an antiapoptotic role, we found a high level of mRNA specific of XAF1, a proapoptotic factor, in A549 cells that are infected by PUUV. In the case of TULV, we found enrichments of proteins involved in biological processes related to RNA binding, to ribosomal function, such as transcriptional and translational events, as well as to ER membrane targeting. Altogether, our data highlighted differences in the way that NSs from different orthohantaviruses may interact with cellular pathways.

## 5. Conclusions

The present study supported a role of hantaviral NSs in antagonizing the IFNβ response. It also demonstrated that other structural hantaviral proteins could participate in this activity. In particular, the GPC of the pathogenic PUUV could be a strong inhibitor acting at both the level of IFNβ production and ISRE activation, while N of PUUV could enhance the IFNβ promoter activity. The spatiotemporal expression of viral proteins will therefore impact interactions with cellular factors during the viral cycle and antiviral response, depending on cell environment. Furthermore, an unexpected role of NSs was observed in infectious contexts, which revealed that IFN in PUUV infected cells was not tardively induced in the absence of a full-length NSs. A plausible scenario, when considering our different observations, could be that PUUV and TULV orthohantaviruses, which did not stimulate an early production of IFN, could do it later. In this regard, we reproducibly observed an increase of IFNβ promoter-driven Luc activity that is induced by the PUUV N protein, which was not seen with TULV N. This could explain the much lower level of IFN that was observed in TULV infected cells as compared to PUUV infected cells. Subsequently, independent of the degree of pathogenicity of orthohantaviruses, GPC could act as major antagonist of this IFN activity. For instance, PUUV NSs could modulate GPC activity by binding to GnCT domain, while, in the absence of NSs, as is the case in PUUVΔNSs variant, GPC would be active to fully inhibit IFN. Another scenario could be that NSs indirectly activated IFN by interaction with a cellular factor, negatively regulating IFN production. In this case, IFN activation would not be impaired in the presence of NSs, while, in the absence of NSs, IFN activity will be repressed by this factor. It could also be argued that IFNβ used in the luciferase assay is not the main cellular antiviral promoter targeted during orthohantavirus infection as sustained by the activation of other IFNs subtypes in infected A549 cells. Homeostasis of interferon activation and inhibition maintaining cellular functions, and its issue during a viral infection, is not easy to predict, since it involves dynamics of networks’ interaction. In this regard, both transcriptomics and proteomics analyses involving NSs protein pointed to a regulated network of interactions. 

Our results support a role of NSs in the IFN-I and IFN-III response, which might be balanced by other hantaviral structural proteins also interfering with these pathways. They also highlight the complexity of interaction among viral proteins, and also between viral proteins and cellular pathways during infection. Combining different approaches at different molecular and cellular levels should help to identify important cellular factors in order to better understand the conceivable different roles of NSs and other viral proteins of orthohantaviruses in counteracting antiviral cellular defense in different hosts. Furthermore, the development of a vole reservoir system is urgently needed in order to understand the evolutionary constraints of hantavirus proteins.

## Figures and Tables

**Table 1:** Genes activated following PUUV Sotkamo wild-type infection of A549 cells.

Gene Name	Log2 Fold ^1^ Change	FDR *p*-Value (<0.05)	Function
XAF1	7.924855639	2.56 × 10^−23^	Positive regulator of apoptosis
IFI44L	7.138156066	3.15 × 10^−15^	Regulator of antiviral response
IFI44	5.614606545	9.61 × 10^−14^	Signaling associated to microtubules
GBP4	4.738550111	9.66 × 10^−6^	IFI GTP binding protein
MX1	4.641241395	1.51 × 10^−12^	IFI, antiviral response
CXCL11	4.49573823	0.000977761	IFI chemokine
RSAD2	4.354185346	5.47 × 10^−7^	IFI, antiviral response, biosynthesis of lipid
OAS2	4.290348742	1.71 × 10^−12^	IFI, inhibition of viral replication
CXCL10	4.182049729	0.012511413	IFNγ induced chemokine
MX2	3.973221891	3.48 × 10^−8^	IFI, antiviral response
CMPK2	3.921882123	1.73 × 10^−8^	Nucleotide synthesis in mitochondria
IFIT1	3.606131209	7.16 × 10^−12^	Inhibition of viral replication and translation
IFI6	3.191888157	3.38 × 10^−9^	IFNα induced, regulation of apoptosis
LAMP3	2.657917461	1.76 × 10^−5^	Lysosome membrane (adaptive immune response)
SIK1	2.656265243	0.0465264	Signaling, metabolism
IFIH1	2.584491344	4.83 × 10^−8^	MDA5, RLR ^2^ signaling
ACKR4	2.537071639	0.021346407	Regulation of immune cells chemotaxis
IFIT3	2.520023935	6.87 × 10^−7^	Adaptor of TBK1 and MAVS
IDO1	2.385541822	0.000502872	Tryptophan catabolism (antimicrobial role)
SAMD9L	2.248439051	2.98 × 10^−8^	Innate immune response to viral infection
DDX60	2.224397585	8.37 × 10^−8^	RNA helicase promoting RLR ^2^ signaling
EPSTI1	2.14797974	3.18 × 10^−5^	Role in STAT1 phosphorylation
IFIT2	2.121715333	6.31 × 10^−6^	Inhibition of viral transcription
SAMD9	2.091207801	6.87 × 10^−7^	Regulation of proliferation and apoptosis
ERBB4	2.083463423	0.047318227	Signaling
DDX58	2.037827005	1.10 × 10^−6^	RIG-I, RNA helicase promoting IFN signaling

^1^ only genes with a log2 fold change higher than 2 as compared to non-infected A549 cells are represented ^2^ RIG-I like receptor.

## Data Availability

The reference for data access is indicated in the Section 2.

## Supplementary Materials

The following Supplementary Materials are available online at https://www.mdpi.com/1999-4915/13/1/140/s1, Figure S1: Golgi localization of Gn in infected cells, Figure S2: Cellular localization of PUUV and TULV NSs proteins coupled to different tags, Figure S3: Effect of viral proteins on TBK1, IRF3 or STAT1 expression and activity. Table S1: (a) Primers used to clone viral sequences in the Gateway system, (b) Primers used for site-directed mutagenesis to validate predicted NoLS regions of NSs, (c) Primers used to quantify mRNA expression of human genes. Table S2: Amino acid sequences of mutant or truncated NSs used in luciferase assay, Table S3: Genes down-regulated in PUUV infected A549 cells.
