# Supplementary material for: Interactions of Viral Proteins from Pathogenic and Low or Non-Pathogenic Orthohantaviruses with Human Type I Interferon Signaling
Source: Viruses. 2021 Jan 19;13(1):140. doi: 10.3390/v13010140 (PMC7835746; doi:10.3390/v13010140)
Supplement: Supplementary file 1 [file viruses-13-00140-s001.pdf]

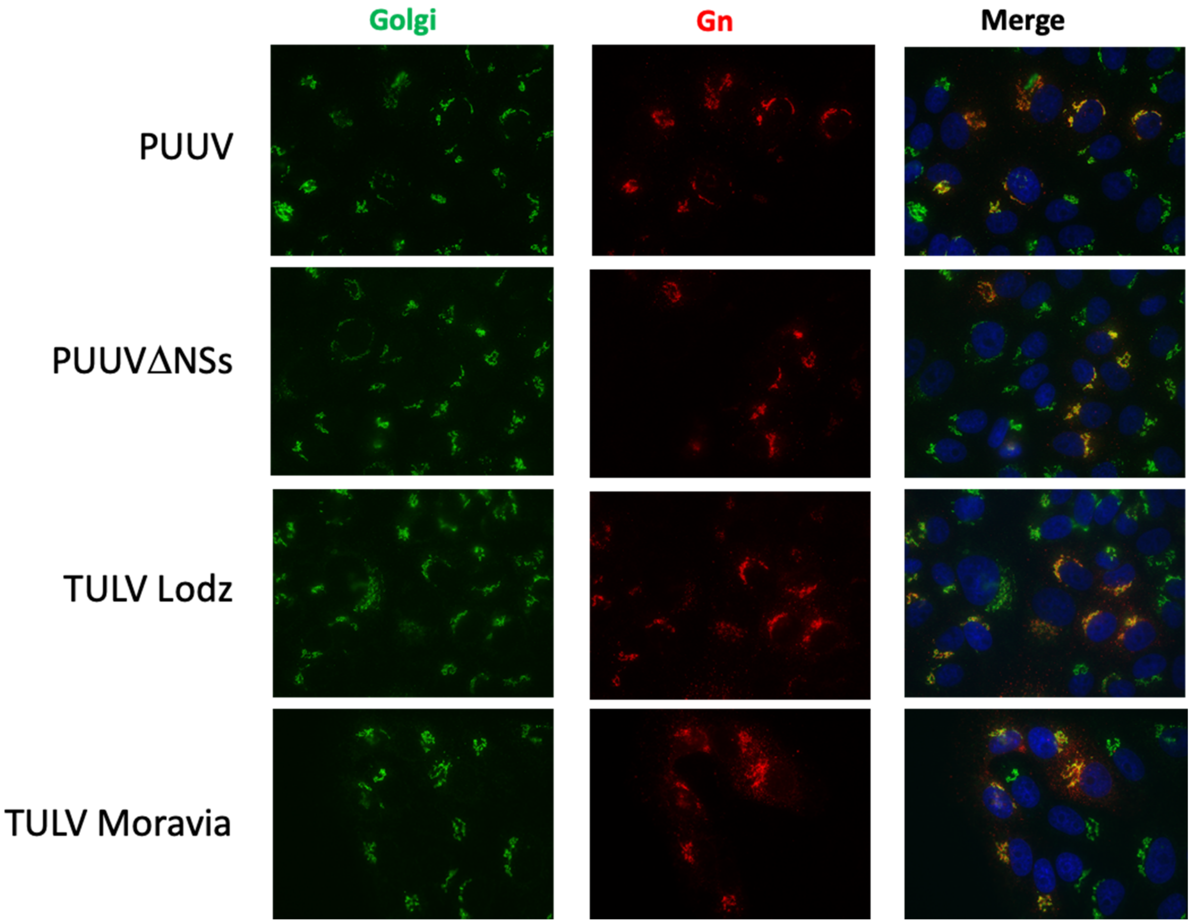

Figure S1. Golgi localization of Gn in infected cells. VeroE6 cells were infected at MOI 0.5 with different wild type or mutant orthohantavirus strains, fixed at day 3 post infection, permeabilized and stained for immunofluorescence with a monoclonal antibody specific to GM130 Golgi marker (green), together with a rabbit serum anti-Gn (red) and then revealed with goat anti-mouse IgG coupled to Alexa 488 or anti-rabbit IgG coupled to Alexa 555 used as secondary antibodies. In the merge panel the nuclei were labelled in blue with DAPI and colocalizing proteins appeared in yellow.

(a)

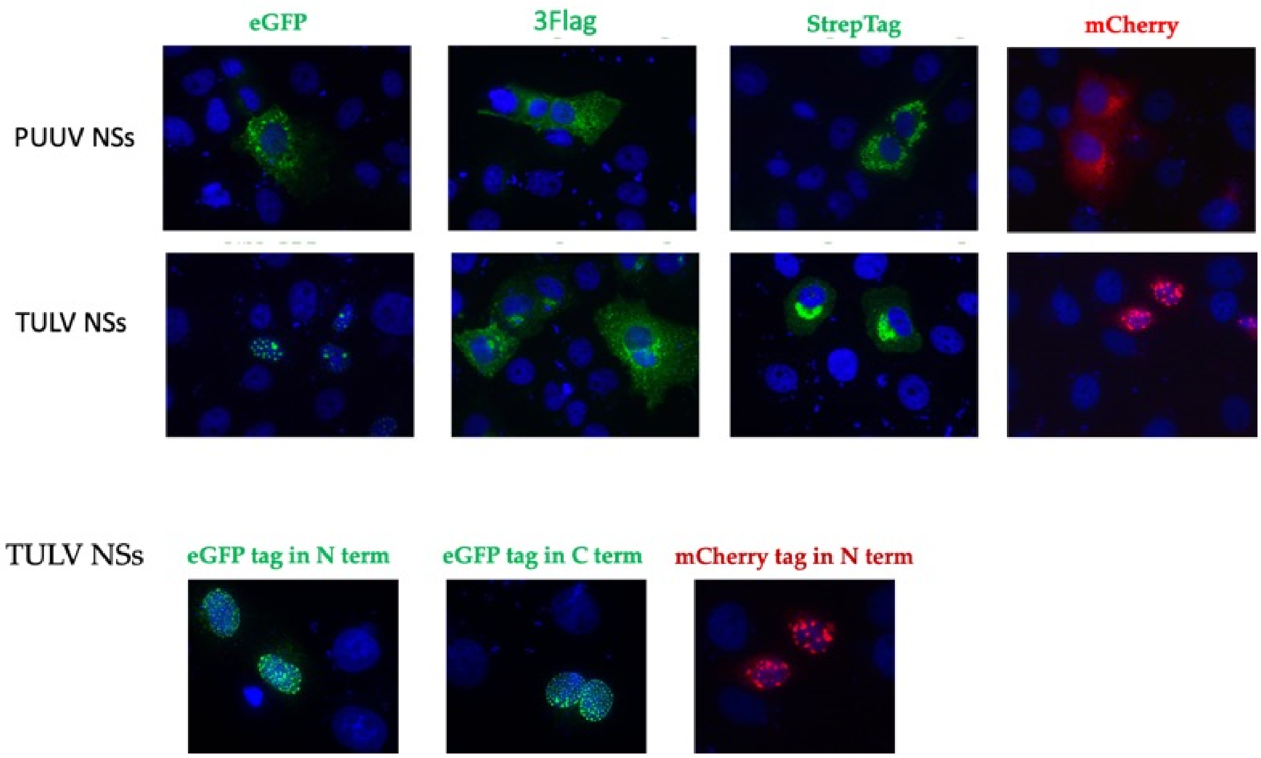

(b)

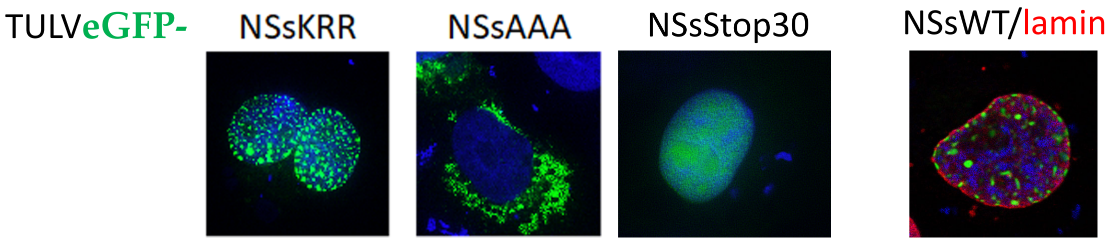

Figure S2. Cellular localization of PUUV and TULV NSs proteins coupled to different tags. VeroE6 cells were transfected with plasmids encoding PUUV or TULV NSs in frame with different tags. In (a) upper panels, eGFP, 3Flag, StrepTag or mCherry were added at the N terminus of PUUV or TULV NSs. In the lower panels the localization of the fluorescent eGFP or mCherry tag added in N-terminus or C-terminus of TULV NSs sequences were compared. In (b) mutant form of eGFP-NSs in which the polar motif was inactivated (NSsAAA) or a stop codon introduced just after the NoLS sequence were transfected in Vero E6 cells to be compared to the localization of wild-type NSs (NSsKRR). Cells were fixed with formaldehyde 24 hours post transfection. Fluorescence analysis was performed using TritonX100 permeabilized cells. The nuclei were labelled with DAPI (blue), eGFP directly fluoresced in green and mCherrry in red. Antibodies specific to the tag were used as primary antibodies to detect 3Flag or StrepTag followed by incubation with goat anti mouse antibody coupled to Alexa 488 (green). Lamin was labeled in red using a monoclonal antibody specific of lamin revealed with a goat anti-mouse IgG coupled to Alexa 555

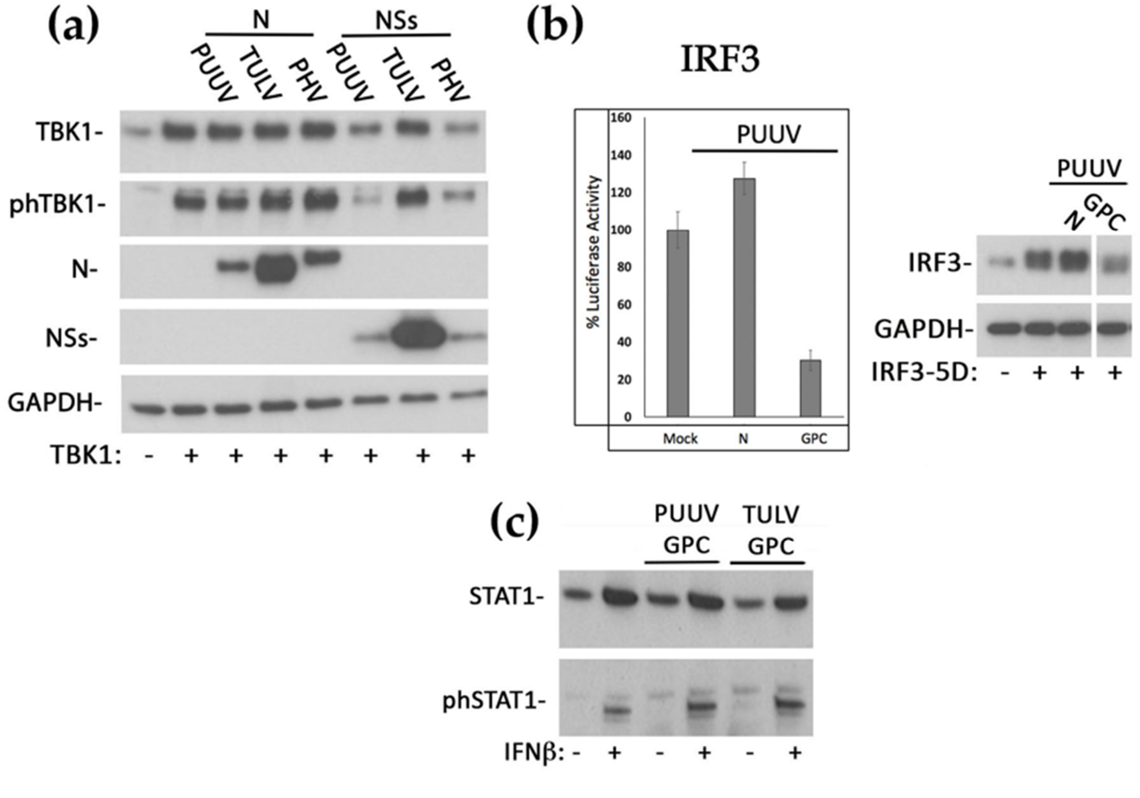

Figure S3. Effect of viral proteins on TBK1, IRF3 or STAT1 expression and activity. a) Expression of TBK1 and phosphorylated TBK1 was analyzed by western blot using specific antibodies for immunodetection of the proteins present in 5 μg of cell lysates from HEK293T cells prepared 24h post transfection with a mix of pCIneo-derived plasmids encoding TBK1 and the N or NSs of the three viruses as compared to transfection with an empty pCIneo plasmid as negative control or plasmid expressing TBK1 alone as positive control. Expression of the N and NSs in the corresponding cell lysates was evaluated using an anti-flag-HRP antibody and anti GAPDH antibody was used to evaluate the homogeneity of the samples. The interference with activated IRF3-5D of the N and GPC from PUUV (b) was measured in the IFN-Luc reporter assay (histogram, left panel) and the expression of IRF3 in transfected samples evaluated by western blot (right panel). In (c) expression of STAT1 and phosphorylated STAT1 in lysates of HEK293T cells, activated or not with IFNβ, at 24h post transfection with GPC constructs of PUUV or TULV (left panel), is shown, by western blot analysis using specific antibodies.

Table S1

(a). Primers for cloning of viral sequences in the gateway system

Virus	Protein	Primer sequence (5′-3′)
**PUUV**	NSs	Fw: ggggacaagtttgtacaaaaaagcaggcttcatgaacagcaacttattgtt
		Rv: ggggaccactttgtacaagaaagctgggtattacatcaaggacatttccatacc
**TULV**	NSs	Fw: ggggacaagtttgtacaaaaaagcaggcttcatgaacagcaaattgtcattgcccggcaaaaacttaaa gatgcagaaaagacggt
		Rv: ggggaccactttgtacaagaaagctgggtattacatcaaggacatttccataccatatcgaaggctt gatct
	GPC	Fw: gatcgggaggtggaggtaccagaaacctttagaactgaaggttg
		Rv: atctgcggccgcaagctttcatttatttttcttataattctgacgac
**PHV**	NSs	Fw: ggggacaactttgtacaaaaaagttggcatgagcagcagcttgtcattgc
		Rv: ggggacaactttgtacaagaaagttggttacatcaaggacatttccatattgg
	GnCT	Fw: ggggacaactttgtacaaaaaagttggcatgaaatatagtaatgactcgaaattc
		Rv: ggggacaactttgtacaaagttgttacctactccgatatctaaacac
	TM1-GnCT[1]	Fw: ggggacaactttgtacaaaaaagttggcatgaaatatagtaatgactcgaaattc
	GnCT-TM2[1]	Rv: ggggacaactttgtacaagaaagttggttacgcacttgcagcccatatgac

[1] for introducing TM1 or TM2 domains the Rv and Fw primers for GnCT amplification were respectively used

(b). Primers used for site-directed mutagenesis to validate predicted NoLS regions of NSs

Virus	Introduced sequence	Primer sequence (5′-3′)
**PUUV**	KRR_{16-18}	Fw: cgtcatctgggtccacttccaccgccttttctgcatccttaagtttttgtc
		Rv: gacaaaaacttaaggatgcagaaaaggcggtggaagtggacccagatgacg
**TULV**	AAA_{16-18}	Fw: cccggcaaaaacttaaagatgcaggcagcagcgtggaagccgacccggatgatgt
		Rv: acatcatccgggtcggcttccacgctgctgcctgcatctttaagtttttgccggg
**PHV**	AAA_{16-18}	Fw: catctgggtccacctccacgctgcggccaagcttccttgagcttctg
		Rv: cagaagctcaaggaagctggccgcagcgtggaggtggacccagtg

(c). Primers used to quantify mRNA expression of human genes

Gene	Forward primer (5′-3′)	Reverse primer (5′-3′)
IFN beta	gtctcctccaaattgctctc	acaggagcttctgacactga
pan IFN alpha	gtgargaaatacttscaaagaatcac	tctcatgatttctgctctgacaa
IFN lambda1	tcctagaccagccccttca	gtgggctgaggctgata
XAF1	gctccacgagtcctactgtg	gttcactgcgacagacatctc
MX1	gtttccgaagtggacatcgca	ctgcacaggttgttctcagc
OAS2	aggtggctcctatggacgg	tttatcgaggatgtcacgttgg
DDX58	tgtgctcctacaggttgtgga	cactgggatctgattcgcaaaa
BST2	cacactgtgatggccctaatg	gtccgcgattctcacgctt
IFI44	ttttcgatgcgaagattcactgg	cctgatgcgttacatgccctt

Table S2. Amino acid sequence of mutant or truncated NSs used in luciferase assay

Mutant	Amino acid sequence[1]
**PUUV_WT**	MNSNLLLPDKNLRMQREQWKWTQMTLIKTHCKPGNKQCQHWRTNSQTTREGWQMLCPGKKWILNLLTRLGLNLMTTSRRDQALGMEMSLM*
**PUUV_1-20**	MNSNLLLPDKNLRMQREQWK---
**PUUV_24-90**	-----------------------MTLIKTHCKPGNKQCQHWRTNSQTTREGWQMLCPGKKWILNLLTRLGLNLMTTSRRDQALGMEMSLM*
**PUUV_stop21**	MNSNLLLPDKNLRMQREQWK*TQMTLIKTHCKPGNKQCQHWRTNSQTTREGWQMLCPGKKWILNLLTRLGLNLMTTSRRDQALGMEMSLM*
**TULV_WT**	MNSKLSLPGKNLKMQKRRWKPTRMMLTRAHYRVDGQLCQHWRTNWQTSRGSLQIWCQVKKWVKSLLTRLGLSRMITSRRDQAFDMEMSLM*
**TULV_24-90**	-----------------------MMLTRAHYRVDGQLCQHWRTNWQTSRGSLQIWCQVKKWVKSLLTRLGLSRMITSRRDQAFDMEMSLM*
**TULV_stop15**	MNSKLSLPGKNLKM*KRRWKPTRMMLTRAHYRVDGQLCQHWRTNWQTSRGSLQIWCQVKKWVKSLLTRLGLSRMITSRRDQAFDMEMSLM*
**TULV_stop30**	MNSKLSLPGKNLKMQKRRWKPTRMMLTRA*YRVDGQLCQHWRTNWQTSRGSLQIWCQVKKWVKSLLTRLGLSRMITSRRDQAFDMEMSLM*
**TULV_AAA**	MNSKLSLPGKNLKMQAAAWKPTRMMLTRAHYRVDGQLCQHWRTNWQTSRGSLQIWCQVKKWVKSLLTRLGLSRMITSRRDQAFDMEMSLM*

[1] Initiating methionine (M_1) of the full-length NSs appears in green; M_{24} potentially used by leaky scanning is coloured in orange; NoLS polar residues (aa_{16-18}) are in violet. Red * correspond to stop codons.

Table S3. Genes down regulated in PUUV infected A549 cells.

Gene ID	Log2 fold change (<-2.5)	FDR P-value (<0.0005)	Gene Name	Description/Function[1]
ENSG00000100276	-5.749047828	0.000411184	RASL10A	GTPase
ENSG00000133475	-4.429845345	0.000209143	GGT2	Glutathione homeostasis
ENSG00000105649	-3.94056695	0.000461217	RAB3A	GTPase vesicle of exocytosis docking
ENSG00000176973	-3.691558342	0.000476243	FAM89B	Negative regulator of TGF signaling
ENSG00000092929	-3.321620848	7.53E-06	UNC13D	Vesicle maturation during exocytosis
ENSG00000115268	-3.276418662	3.18E-06	RPS15	Ribosomal protein
ENSG00000165887	-3.256162331	8.66E-06	ANKRD2	Muscle ankyrin repeat protein
ENSG00000167799	-3.201243179	0.000309949	NUDT8	Nucleoside diphosphate hydrolysis
ENSG00000186765	-3.163130811	1.37E-05	FSCN2	Actin bundling protein
ENSG00000176092	-3.145393666	0.000165932	AIM1L	Lectin-carbohydrate binding
ENSG00000214026	-3.104268277	0.000355407	MRPL23	Mitochondrial ribosomal protein
ENSG00000168481	-3.093601941	2.49E-05	LGI3	Role in exocytosis
ENSG00000175793	-3.073964722	5.47E-06	SFN	Regulates cell cycle, ubiquitination, degradation
ENSG00000176101	-3.030504643	5.39E-08	SSNA1	Nuclear autoantigen
ENSG00000173267	-3.03457469	1.64E-05	SNCG	Neurofilament integrity, protein secretion
ENSG00000178814	-3.001632855	0.000107822	OPLAH	Glutamate synthesis
ENSG00000166166	-2.970492526	8.66E-06	TRMT61A	tRNA methyltransferase
ENSG00000167775	-2.964668743	7.59E-07	CD320	Vitamin B12 receptor, lymphocyte proliferation
ENSG00000169738	-2.956701598	9.89E-06	DCXR	Glucose metabolism
ENSG00000187840	-2.915166258	3.18E-05	EIF4EBP1	Repressor of translation by EIF4E binding
ENSG00000131668	-2.929944238	0.000160679	BARX1	Homeobox transcription factor
ENSG00000167680	-2.933701352	8.89E-06	SEMA6B	Nervous system development
ENSG00000100092	-2.90360021	5.47E-06	SH3BP1	Addressing of secretory vesicles to the PM
ENSG00000100949	-2.876318323	3.72E-05	RABGGTA	Transfer of geranylgeranyl on Rab proteins
ENSG00000130005	-2.884404074	6.45E-05	GAMT	Methyltransferase
ENSG00000172889	-2.888940965	7.45E-06	EGFL7	Endothelial cell adhesion to ECM, angiogenesis
ENSG00000197903	-2.893236477	9.68E-06	HIST1H2BK	Nucleosome component
ENSG00000196923	-2.843285317	6.33E-06	PDLIM7	Scaffold adapter of kinase (LIM) and actin
ENSG00000160446	-2.828662202	9.66E-06	ZDHHC12	Probable palmytoyltransferase
ENSG00000130255	-2.820244525	1.31E-05	RPL36	Ribosomal protein
ENSG00000131669	-2.820354274	1.15E-05	NINJ1	Cell adhesion molecule
ENSG00000103363	-2.802856763	1.95E-05	TCEB2	Transcription elongation factor
ENSG00000214063	-2.806424423	7.57E-07	TSPAN4	Signaling by complexing with integrins
ENSG00000174775	-2.807530523	7.32E-06	HRAS	Ras proto-oncogene GTPase
ENSG00000169750	-2.81265636	0.000192992	RAC3	Lipid vesicle transport
ENSG00000196924	-2.785564148	7.57E-07	FLNA	Cytoskeletal remodelling
ENSG00000142544	-2.795062154	0.000235973	CTU1	Adenylation of tRNA
ENSG00000165886	-2.78120402	0.000310491	UBTD1	Ubiquitination and degradation of MDM2
ENSG00000197114	-2.782102358	0.000394068	ZGPAT	Transcription repressor of EGFR
ENSG00000124664	-2.774699294	4.02E-05	SPDEF	Transcriptional activator
ENSG00000142235	-2.760336501	1.47E-05	LMTK3	Protection against proteasome degradation

ENSG00000160932	-2.765011077	1.50E-05	LY6E	Lymphocyte antigen, T cell development
ENSG00000110711	-2.765544874	3.01E-05	AIP	IL12 signaling pathway
ENSG00000126458	-2.765872058	4.32E-05	RRAS	Angiogenesis. cellular adhesion
ENSG00000174996	-2.741543009	7.32E-06	KLC2	Microtubule-associated organelle transport
ENSG00000130193	-2.747424514	9.40E-06	THEM6	Thioesterase
ENSG00000131584	-2.740390083	2.18E-07	ACAP3	Regulation of GTPase activity
ENSG00000197785	-2.734613298	6.64E-06	ATAD3A	Apoptosis and innate immune response
ENSG00000177943	-2.719773073	0.000163052	MAMDC4	Sorting and selective transport
ENSG00000185133	-2.721208566	0.000112412	INPP5J	Inositol phosphate metabolism
ENSG00000187486	-2.70213566	0.000438876	KCNJ11	Ion channel protein
ENSG00000175756	-2.702933132	3.22E-05	AURKAIP1	mRNA processing, translation
ENSG00000177697	-2.71222339	2.06E-05	CD151	Signaling by complexing with integrins
ENSG00000108479	-2.715067675	6.64E-06	GALK1	Metabolism of galactose
ENSG00000177106	-2.698839043	2.36E-06	EPS8L2	Actin organization
ENSG00000167716	-2.699551244	1.99E-05	WDR81	Regulator of PI3K, endolysosomal trafficking
ENSG00000261236	-2.699591898	1.37E-05	BOP1	Maturation of ribosomal RNA
ENSG00000130489	-2.693754048	7.18E-05	SCO2	COX assembly factor metallochaperone
ENSG00000014164	-2.696627325	1.57E-05	ZC3H3	PolyA mRNA nuclear export
ENSG00000163795	-2.691835933	9.20E-06	ZNF513	Transcriptional regulator
ENSG00000101210	-2.677924259	2.64E-05	EEF1A2	Translation elongation factor
ENSG00000030582	-2.680119841	6.09E-05	GRN	Inflammatory response, protein folding, apoptosis
ENSG00000179271	-2.680581691	1.31E-06	GADD45GIP1	Cell cycle regulation
ENSG00000168528	-2.683470776	0.000275316	SERINC2	Serine transporter
ENSG00000054148	-2.683782226	2.06E-05	PHPT1	T cell signaling. cytoskeleton organization
ENSG00000161999	-2.674902804	8.66E-06	JMJD8	Regulator of TNF-induced NFKB signaling
ENSG00000167994	-2.663986811	3.01E-05	RAB3IL1	Guanine nucleotide exchange factor of RAB3A
ENSG00000188130	-2.666907396	1.13E-05	MAPK12	Ser/Thr kinase, extracellular signal transduction
ENSG00000128591	-2.668768884	8.76E-05	FLNC	Cross-linking of actin filaments
ENSG00000188375	-2.660395116	1.93E-05	H3F3C	Nucleosome component
ENSG00000167779	-2.646529347	2.99E-05	IGFBP6	Regulation of IGF signaling pathway
ENSG00000167468	-2.654355441	1.37E-05	GPX4	Protection against oxidative damage
ENSG00000128185	-2.641080676	5.45E-05	DGCR6L	Neural development
ENSG00000176978	-2.645996247	1.90E-05	DPP7	Proteolysis, neutrophil degranulation
ENSG00000160867	-2.622690056	1.99E-05	FGFR4	FGF signaling
ENSG00000147813	-2.6246977	1.68E-05	NAPRT	Nicotinate phosphoribosyltransferase
ENSG00000105669	-2.594360037	0.000115347	COPE	Budding from Golgi membranes
ENSG00000242372	-2.597233424	8.89E-06	EIF6	Translation initiation factor
ENSG00000100241	-2.597463107	2.05E-05	SBF1	GEF activating Rab28
ENSG00000188372	-2.598825968	1.96E-08	ZP3	Extracellular matrix glycoprotein
ENSG00000129932	-2.601065297	0.000185688	DOHH	Post-translation modification
ENSG00000159363	-2.611417087	5.65E-05	ATP13A2	ATPase transporter
ENSG00000177595	-2.611748997	8.20E-05	PIDD1	Adaptor, cell death-related signaling processes
ENSG00000161016	-2.61445624	8.89E-06	RPL8	Ribosomal protein
ENSG00000184363	-2.616692132	7.92E-05	PKP3	Cell adhesion
ENSG00000107331	-2.587639388	2.89E-05	ABCA2	ATP binding cassette transporter
ENSG00000160211	-2.588188345	1.47E-05	G6PD	Glucose metabolism
ENSG00000205277	-2.589402099	7.44E-06	MUC12	Epithelial cell signaling
ENSG00000174917	-2.584422354	0.000101042	C19orf70	Inner mitochondrial membrane organization
ENSG00000143537	-2.574530306	8.66E-06	ADAM15	Angiogenesis, regulation of cell adhesion
ENSG00000174886	-2.575281029	2.07E-05	NDUFA11	Mitochondrial respiratory chain assembly
ENSG00000142798	-2.576265786	5.47E-06	HSPG2	Angiogenesis
ENSG00000100379	-2.577309325	0.000252523	KCTD17	Positive regulator of ciliogenesis
ENSG00000169976	-2.578935249	0.000169175	SF3B5	mRNA processing
ENSG00000065268	-2.579371361	6.00E-05	WDR18	Cycle progression, signal transduction, apoptosis
ENSG00000173599	-2.565069184	1.12E-05	PC	Gluconeogenesis, lipid metabolism
ENSG00000167657	-2.565293239	3.23E-05	DAPK3	Induction of apoptosis
ENSG00000101197	-2.565380407	0.000166209	BIRC7	Member of apoptosis family inhibitor
ENSG00000100079	-2.565923081	0.000156269	LGALS2	Galectin 2
ENSG00000170889	-2.567468036	3.67E-06	RPS9	Small ribosomal protein subunit
ENSG00000062822	-2.5743698	6.64E-06	POLD1	DNA replication and repair
ENSG00000032444	-2.557691174	4.98E-05	PNPLA6	Lipid metabolism
ENSG00000198931	-2.560927959	4.76E-05	APRT	Purine metabolism
ENSG00000123143	-2.553559717	1.25E-05	PKN1	Transcription regulation
ENSG00000132017	-2.55390037	1.37E-05	DCAF15	Ubiquitination involved in degradation

ENSG00000198055	-2.554161062	1.75E-05	GRK6	Cell chemotaxis, receptor signaling
ENSG00000185187	-2.546973187	2.07E-05	SIGIRR	Negative regulator of TLR and IL-1R
ENSG00000071894	-2.547372687	9.29E-05	CPSF1	mRNA metabolism
ENSG00000183751	-2.550202757	9.18E-06	TBL3	rRNA processing
ENSG00000130706	-2.550409606	1.03E-05	ADRM1	Component of proteasome ubiquitin receptor
ENSG00000105701	-2.54535512	6.64E-06	FKBP8	Protein folding and trafficking
ENSG00000183617	-2.53524744	4.79E-05	MRPL54	Mitochondrial ribosomal protein
ENSG00000185803	-2.539851568	0.000119641	SLC52A2	Vitamin B2 transporter
ENSG00000141959	-2.542047853	7.80E-05	PFKL	Glycolysis
ENSG00000187091	-2.531485269	4.15E-05	PLCD1	Signal transduction in lipid metabolism
ENSG00000064932	-2.523172344	5.73E-05	SBNO2	Pro-inflammatory coregulator, repression of NFKB
ENSG00000100258	-2.523192277	0.000175522	LMF2	Protein maturation and transport
ENSG00000167114	-2.523247215	4.60E-05	SLC27A4	Fatty acid transport
ENSG00000179950	-2.524722701	5.14E-05	PUF60	Apoptosis and transcription regulation
ENSG00000102977	-2.525158822	7.44E-06	ACD	Involved in telomere functions
ENSG00000076924	-2.52524537	1.30E-05	XAB2	mRNA processing
ENSG00000126254	-2.525512968	7.44E-06	RBM42	mRNA splicing
ENSG00000142327	-2.526712071	6.75E-05	RNPEPL1	Proteolysis
ENSG00000179091	-2.519802651	7.32E-06	CYC1	Mitochondrial respiratory chain
ENSG00000172354	-2.515342608	6.06E-06	GNB2	Receptor signaling
ENSG00000179403	-2.517101883	8.14E-05	VWA1	Matrix assembly
ENSG00000179115	-2.513177599	8.89E-06	FARSA	Translation
ENSG00000175467	-2.514387166	1.03E-05	SART1	mRNA processing
ENSG00000186010	-2.51081367	0.000119608	NDUFA13	Mitochondrial respiratory chain
ENSG00000130475	-2.505159354	1.03E-05	FCHO1	Clathrin mediated endocytosis
ENSG00000141985	-2.506799156	3.67E-06	SH3GL1	Endocytosis
ENSG00000184967	-2.508435915	0.000487721	NOC4L	RNA processing
ENSG00000184281	-2.50866512	6.64E-06	TSSC4	Tumor suppressor candidate

[1] functions are highlighted as follows:
cell trafficking, cell cycle and organization;
replication, transcription, translation;
cell signaling, degradation apoptosis.
